# Novel Isoindigo-Based Organic Semiconductors End Capped with 1,1-Dicyanomethylene-3-Indanone: Effect of the Bromination and Position of Bromine Substituents on the Chemical–Physical and Electrical Properties

**DOI:** 10.3390/molecules30183672

**Published:** 2025-09-09

**Authors:** Fabio Mocerino, Mario Barra, Fabio Borbone, Antonio Carella, Roberto Centore, Fabio Chiarella, Alessandro Landi, Andrea Peluso

**Affiliations:** 1 Dipartimento di Scienze Chimiche, Università Degli Studi di Napoli Federico II, Complesso Universitario di Monte Sant’Angelo, via Cintia 21, 80126 Napoli, Italy; fabio.mocerino@unina.it (F.M.); fabio.borbone@unina.it (F.B.); roberto.centore@unina.it (R.C.); 2 CNR—Institute for Superconductors, Innovative Materials and Devices, SPIN, Piazzale Tecchio 80, 80125 Napoli, Italy; mario.barra@spin.cnr.it (M.B.); fabio.chiarella@spin.cnr.it (F.C.); 3 Dipartimento di Chimica e Biologia, Università di Salerno, Via Giovanni Paolo 132, 84084 Fisciano, Italy; alelandi1@unisa.it (A.L.); apeluso@unisa.it (A.P.)

**Keywords:** n-type organic semiconductors, isoindigo derivatives, synthesis, DFT, OFETs

## Abstract

We report here on the synthesis and characterization of three novel isoindigo (II)-based organic semiconductors. The three dyes are based on an electron acceptor II core, symmetrically linked to two 3-octylthiophene donor rings; this common fragment, easily synthesizable, is end-capped with three different auxiliary electron acceptor groups, 1,1-Dicyanomethylene-3-Indanone (**IDM**) and two derivatives of it, bearing a bromine atom in position 5 or 6 of the **IDM** ring. The effect of the bromination and of the position of the bromine atom on the chemical–physical and electrical properties of the compounds were examined by means of thermal, optical, and electrochemical analysis; the electronic properties were investigated in more details at the DFT level. The novel compounds were used as active layers in organic field effect transistors: all the II derivatives were n-type unipolar semiconductors with electron mobilities ranging between 10^−3^ and 10^−4^ cm^2^/V∙s.

## 1. Introduction

**Scheme 1:**
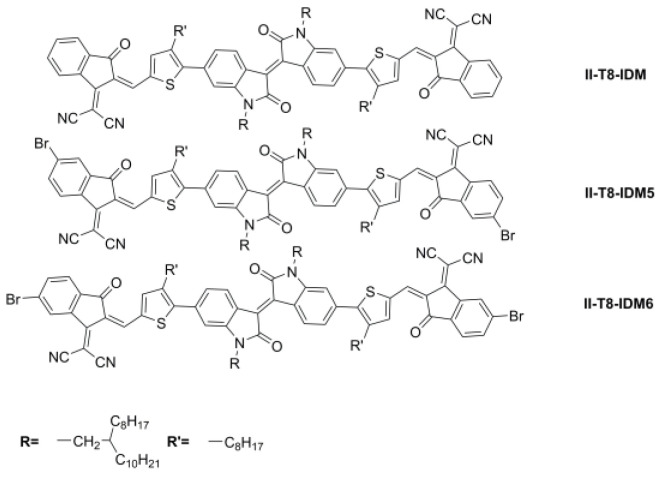
Molecular structure of the synthesized **II**-based organic semiconductors.

**Scheme 2:**
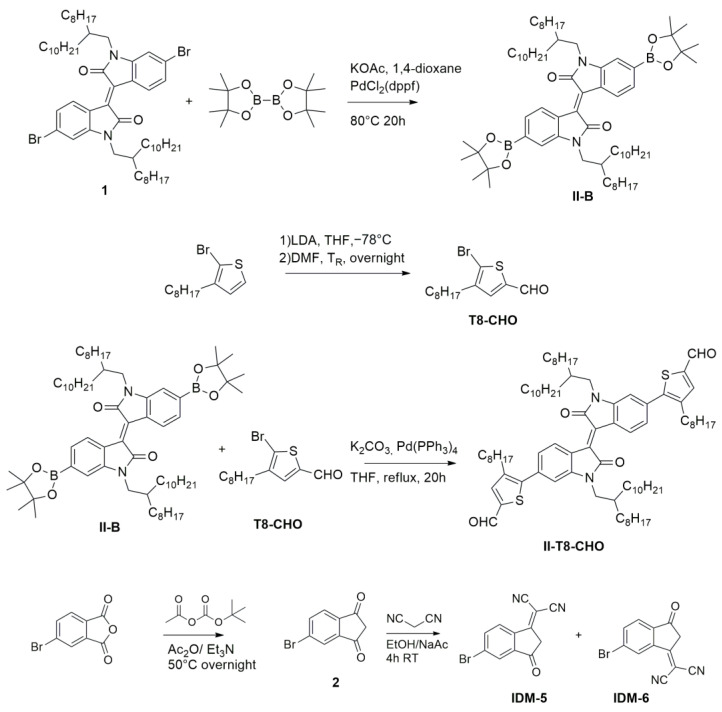
Synthetic pathway for the preparation of the precursors of **II**-based materials here reported.

Interest in organic semiconductors (OSCs) has grown enormously over the last twenty years due to their peculiar mechanical and processing properties [1,2,3,4]. The possibility of processing them from a solution and their compatibility with flexible substrates [5] have opened the way to several new applications, such as wearable devices [6,7], biosensors [8], flexible OLED displays [9], stretchable transistors [10], etc., that would not be possible with materials traditionally used in electronics (e.g., silicon) [11,12,13,14]. Historically, p-type OSCs emerged first, and the number of works regarding these materials is significantly higher than those related to n-type analogous varieties [15,16,17]. The development of new high-performance n-type OSCs, however, is essential for several types of applications, first and foremost for the fabrication (together with their p-type counterpart) of complementary logic circuits that represent the reference technology in contemporary electronic circuitry [18,19]. These types of materials is also of fundamental importance in the field of organic photovoltaics, where the active layer is typically made up of a blend of a p-type (donor) and n-type (acceptor) compounds in a type of configuration known as a bulk heterojunction solar cell [20]. In this sector, in recent years, we have witnessed a significant development of new organic semiconductors of the n-type non-fullerene acceptors (NFA) [21,22,23,24] that have contributed to bring the efficiency of bulk heterojunction solar cells to over 20% [25]. It is also important to underline that NFAs are currently finding use in perovskite solar cells as electron transporting materials [26,27,28]. It is clear from what has been said so far that the interest in developing new n-type organic semiconductors is very high. Among NFAs, the ITIC and Y6 classes should be particularly mentioned: ITIC derivatives [29,30] are based on a DD’D fused unit (indacenodi-thieno [3,2-b]thiophene) as a central core, symmetrically linked to terminal acceptor groups, while the Y6 derivatives [31,32,33] are based on a DAD-type central fused ring (dithienothiophene-[3,2-b]-pyrrolobenzothiadiazole), with benzo-thiadiazole in the center. Also, in this case, the molecular backbone is ended with terminal acceptor groups. For both classes of NFAs, the typical end acceptor group is 2-(3-Oxo-2,3-dihydroinden-1-ylidene)-malononitrile (**IDM**) or some derivatives of it [29,32]. Exploring cores based on single-bond-linked units rather than on large, fused structures could be useful in reducing the complexity of the synthesis [34]. In this context, we recently synthesized some isoindigo (**II**) derivatives, based on a di-thienyl(furyl)-isoindigo core symmetrically linked to terminal **IDM** units, displaying interesting n-type properties [35]. As a development of the previous work, we report here on the synthesis of three novel II derivatives, whose molecular structure is shown in Scheme 1. Two main structural modifications have been implemented in the present work. First of all, the donor thiophene rings linked to the **II** core were functionalized with a linear octyl tail; this modification is expected to inductively increase the donor strength of thiophene rings and at the same time, to enhance solubility. The second proposed change is the modification of the **IDM** end group by introducing a bromine atom in position 5 or 6 of the indandione unit. This functionalization is expected to increase the acceptor strength of the **IDM** group and consequently, to also affect the LUMO energy of the molecules. Halogenated derivatives of **IDM** are obtained in a mixture of two isomers, and typically, this mixture is coupled to the central core to obtain derivatives that are not isomerically pure. In this case, we instead purified the brominated **IDM** precursor to obtain the pure isomer (**IDM-5** or **IDM-6**, see Scheme 2), and the final materials are therefore precisely defined regarding their molecular constitution. Consequently, we could investigate the effect of the bromine position on the chemical–physical and electrical properties of the new materials.

All the synthesized materials were carefully characterized in terms of their thermal, optical, and electrochemical properties. A thorough computational analysis was performed to better investigate electronic properties of the II derivatives. Thin films of the new molecules were finally used as active layers of organic field-effect transistors (OFETs), and electrical characterization of the fabricated OFETs provided information about their charge transport behavior and charge carrier mobility.

## 2. Results and Discussion

### 2.1. Synthesis and Thermal Characterization

**Scheme 3:**
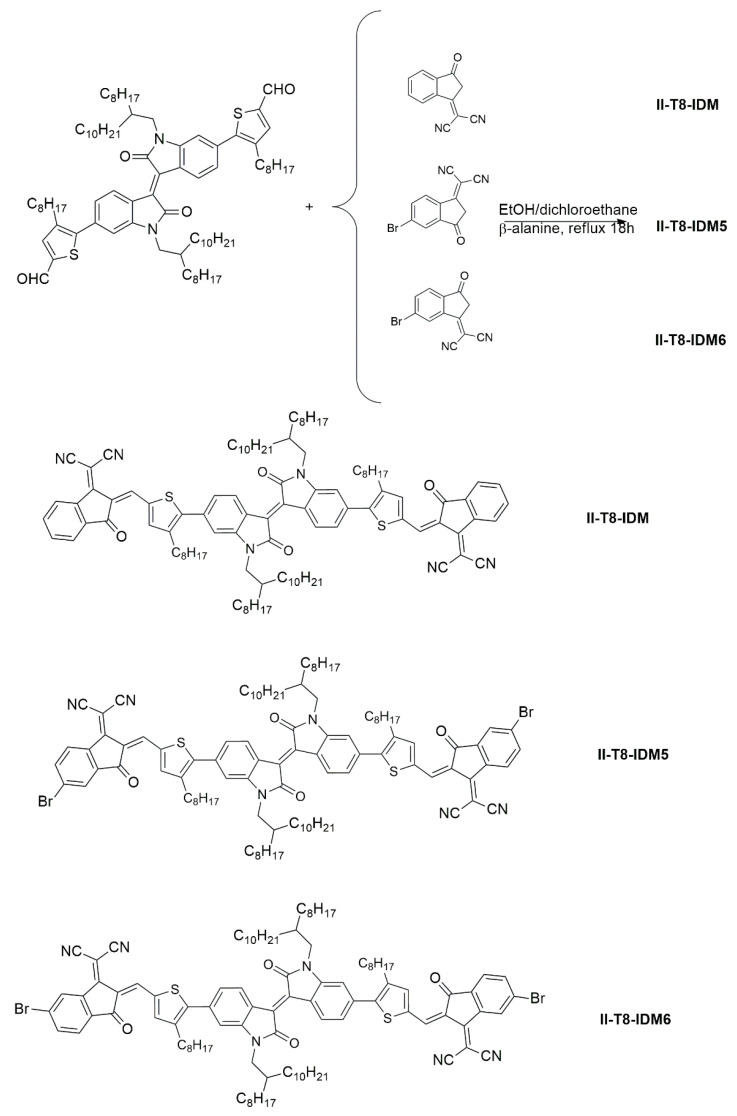
Synthesis of the final compounds.

The three new **II**-based OSCs have been prepared through a multistep synthetic procedure, as schematically shown in Scheme 2 and Scheme 3. 

The synthesis of the **II** core and its functionalization with long and branched alkyl tails (2-octyldodecyl group) was achieved following the procedure described in our previous work [35]. A di-boronated derivative (**II-B**) of this precursor was then synthesized by means of the well-known Miyaura borylation reaction. Compound **T8-CHO** was synthesized, starting from the commercial 2-bromo-3-octylthiophene, by reacting it at −78 °C with lithium diisopropylamide (LDA) and then N,N-dimethylformamide (DMF) as a formylating agent. Suzuki coupling between **II-B** and **T8-CHO** afforded the di-formyl precursors **II-T8-CHO** in a good yield. Concerning the end acceptor, **IDM** was synthesized as previously reported [36]. The mono-brominated variant of **IDM** was prepared in two steps using the commercial 4-bromophthalanhydride as a starting compound, following a procedure reported in the literature [37]. In this way, a mixture of two isomers (approximately in a 1/1 ratio) was obtained, where the inserted bromine atom functionalizes position 5 **(IDM-5**) or 6 (**IDM-6**). We managed to separate the mixture into the pure isomers by exploiting their selective solubility in different solvents; the mixture was treated with acetonitrile at room temperature overnight, and after filtration, we obtained an **IDM-5**-rich precipitate and an **IDM-6**-rich mother liquor. The **IDM-6**-rich fraction was dried and then recrystallized from 1,2-dichloroethane, obtaining pure **IDM-6**. In this process we obtained crystals of suitable size for XRD analysis; by solving the structure using this technique, we also confirmed that the isolated isomer was indeed **IDM-6** (see Figure S1). The **IDM-5** rich precipitate was washed one more time with acetonitrile and then recrystallized by CHCl_3_, obtaining pure **IDM-5**. Finally, the three end acceptors were reacted with **II-T8-CHO** in a Knoevenagel condensation reaction to obtain the desired **II**-based final compounds (respectively, **II-T8-IDM**, **II-T8-IDM5** and **II-T8-IDM6**), as shown in Scheme 3. The chemical identity of all the compounds was primarily confirmed by means of 1H, and then by 13C NMR, Mass Spectroscopy, and FTIR analysis (see Figures S2–S26). The FTIR spectra of the three target molecules are characterized by the presence of a signal at around 2200 cm^−1^, characteristic of the stretching of the triple carbon nitrogen bond of the cyano group (this signal was obviously absent in the precursor aldehyde). Other distinctive IR signals for the synthesized isoindigo-based OSCs are the group of peaks at around 3000 cm^−1^ (aliphatic and aromatic C-H stretching), the C=O stretching just below 1700 cm^−1^, and the aromatic C=C stretching at 1600 cm^−1^. No clear signals related to C–Br bond emerged from FTIR analysis. This is not surprising because the frequency of this bond is expected to lie in the crowded fingerprint zone of the spectrum The actual presence of two bromine atoms in **II-T8-IDM5** and **II-T8-IDM6** was instead confirmed by MS analysis: in their MS spectra, the presence of [M]^+^, [M + 2]^+^, and [M + 4]^+^-related signals (see Figures S21 and S25 and the Materials and Methods Section) are in fact distinctive of dibrominated organic compounds as a consequence of the similar relative abundance of ^79^Br and ^81^Br isotopes.

A DSC analysis was performed on the three II-based OSCs; they are all characterized by a polymorph behavior with at least one solid-to-solid transition, as shown in Figures S27–S29. The melting temperatures are reported in Table S2. **II-T8-IDM5** shows by far the higher melting point, 237 °C, which is 24 °C higher than that of its isomer **II-T8-IDM6**. The non-brominated derivative **II-T8-IDM** displays the lowest melting temperature (202 °C); a direct comparison between the last compound and the similar system **II-T-IDM** reported in our previous paper [35] highlights a significant melting point decrease (202 vs. 275 °C). The structural difference between these two derivatives is the octyl tail functionalizing the thiophene ring in **II-T8-IDM**, which is absent in **II-T-IDM**. The dramatic impact of the octyl chain on the melting point could probably be associated with a decrease in planarity, determined by an expected torsion around the bond linking the **II** core and the thiophene ring. The functionalization with the octyl chain also impacts the solubility of the new dyes: while for the previously reported **II-T8-IDM**, solubility was lower than 5 mg/mL [35], the new dyes can be solved (in chloroform) at concentrations of at least 10 mg/mL.

### 2.2. Optical Characterization

**Figure 1:**
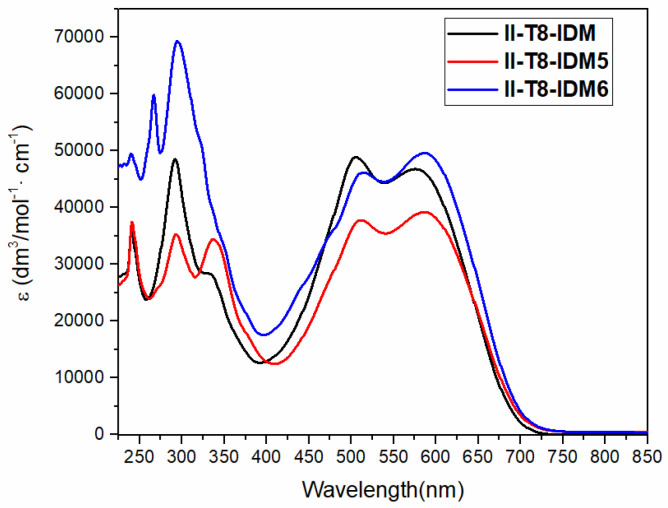
UV–Vis absorption spectra in chloroform solution for the three **II**-based OSCs.

The II-based OSCs were optically characterized in chloroform solution; the UV–Vis absorption spectra are shown in Figure 1, and the main optical features are summarized in Table 1; in the visible region of the spectrum, all three dyes show two distinct absorption peaks of similar intensity at around 500 and 580 nm, imparting a violet color to the solutions. A clear red shift of the higher wavelength peaks is observed upon bromination of the end **IDM** acceptor group (see Table 1). In the UV–Vis region of the spectrum, we can observe two main peaks of similar intensity, i.e., in the case of **II-T8-IDM5**, at around 290 and 300 nm. Instead, the optical feature at 290 nm prevails over that at 330 nm in **II-T8-IDM**. In the case of **II-T8-IDM6**, we can see a very strong absorption at around 290 nm, with the peak at around 330 nm turning to a shoulder. Quantitatively, **II-T8-IDM** and **II-T8-IDM6** present comparable molar extinction coefficients in the visible part of the spectrum (although with an inversion of the relative intensity between the two peaks), while the **II-T8-IDM5** photon harvesting ability is slightly weaker.

**Figure 2:**
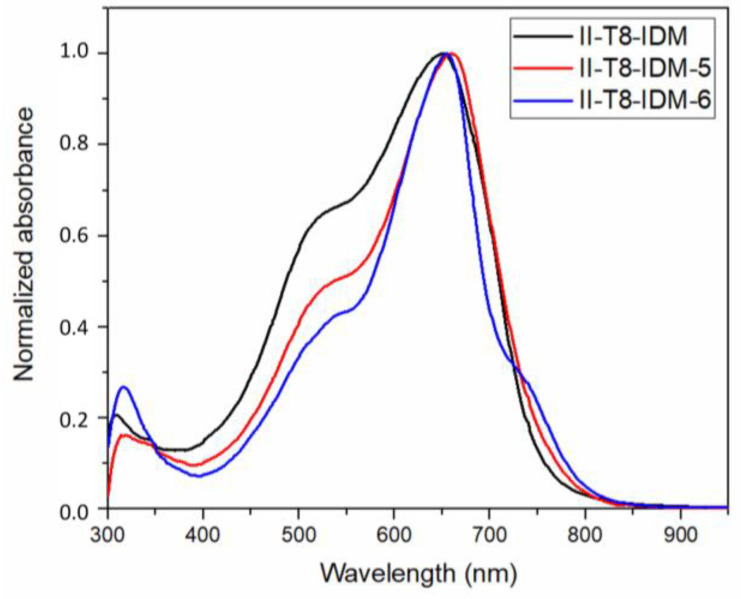
UV–Vis absorption spectra of **II**-based OSCs as thin films spin-coated from 1,1-2,2-tetrachloroethane.

The optical characterization was performed on the dyes’ thin films as well (see Figure 2 and Table 2). The films were prepared via spin coating from 1,1-2,2-tetrachloroethane solution, following the procedure used for the preparation of OFETs (vide infra). In Figure 2, the optical absorption spectra of the films are reported. The absorption of the three compounds moves, in the solid state, to higher wavelengths. For all the **II** derivatives, it is possible to observe a shoulder at lower wavelengths. Only in the case of **II-T8-IDM6**, a further shoulder at higher wavelength appears upon annealing at temperatures higher than 120 °C. The optical bandgap was graphically determined by the optical spectra (Figures S30–S32): **II-T8-IDM** and **II-T8-IDM5** possess similar bandgap values, while a lower bandgap is observed for **II-T8-IDM6**.

### 2.3. DFT Analysis

To acquire more information about the electronic structure of the synthesized dyes, we have performed a computational analysis at the PBE0/6-31 + g* level, including solvent effects (chloroform) through the polarizable continuum model. As commonly executed in this kind of study, the side alkyl chains have been modeled as a methyl groups [38].

All the molecules have been optimized in their minimal energy structures (optimized molecular geometries for the three compounds are reported in Figures S33–S35 in the Supplemental Materials section), where they show a slightly twisted conformation.

**Figure 3:**
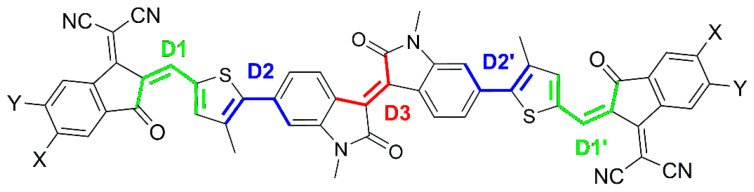
Schematic representation of the dyes’ torsional patterns.

The molecule can be divided into three fragments (see Figure 3 and Table 3), connected by the three torsion angles D1, D2, D3 around the bond linking the external fragments with the thiophene ring, the bond between thiophene and the core, and the link between the two oxindole portions of the core, respectively. The dihedral angles D1 and D2 appear twice (D1′ and D2′) and display very similar values, owing to the aforementioned symmetry. As already hypothesized in a previous paragraph, the dihedral angle D2 (D2′) is sufficient (see Table 3) to induce a significant disruption of the overall planarity of the molecule.

The predicted absorption spectra are reported in the Supplemental Materials (Figures S36–S38), together with a comparison with the experimental ones. Wavelengths and oscillator strengths (larger than 0.15) between the ground state orbitals to the excited state are reported in Table S2. The theoretical spectrum in the hundred-excitation range explored by TD-DFT is made up of three principal bands, the highest basically corresponding to the HOMO -> LUMO transition. A good qualitative accord with experimental behavior is observed, even though absorption wavelengths are overestimated by about 60 nm. In Table 4, we report information about these main transitions, the dipole moments of the ground state and of the first excited state, and the optical band gap obtained from the TD-DFT vertical transition (E_g_). The latter results are in good agreement with the experimental values, thus validating the calculation and the choice of the density functional.

The brominated derivatives are characterized by a higher dipole moment value (see Table 4) as compared to that of the not-brominated **II-T8-IDM**. In particular the polarity of the molecule increases significantly when the bromine atom functionalizes position 6 of the **IDM** ring. Indeed, although Br atoms are symmetrically placed on both terminal acceptor units, the inherent helical distortion of the molecule prevents full cancellation of their local dipole contributions. The resulting spatial asymmetry and out-of-plane orientation of the electron density leads to an increase in the net dipole moment, particularly along the axis perpendicular to the molecular plane.

**Figure 4:**
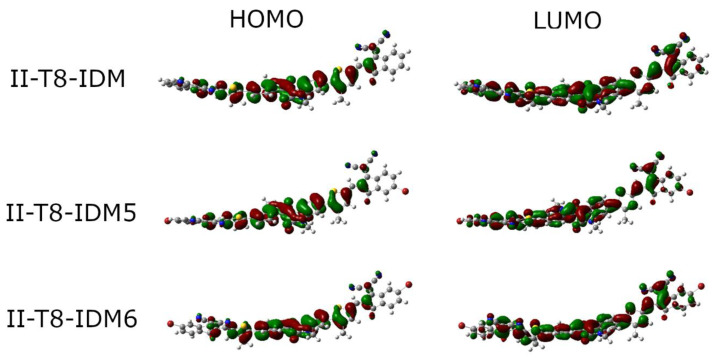
Computed isodensity surfaces of HOMO (left panel) and LUMO (right panel) of the dyes analyzed in this work.

For all the chromophores, a consistent decrease in the overall dipole moment values is observed when passing from ground to excited state: upon transition to the excited state electron density experiences only a slight displacement toward the end acceptor groups. HOMO and LUMO density distribution is shown in Figure 4.

### 2.4. Electrochemical Characterization

**Figure 5:**
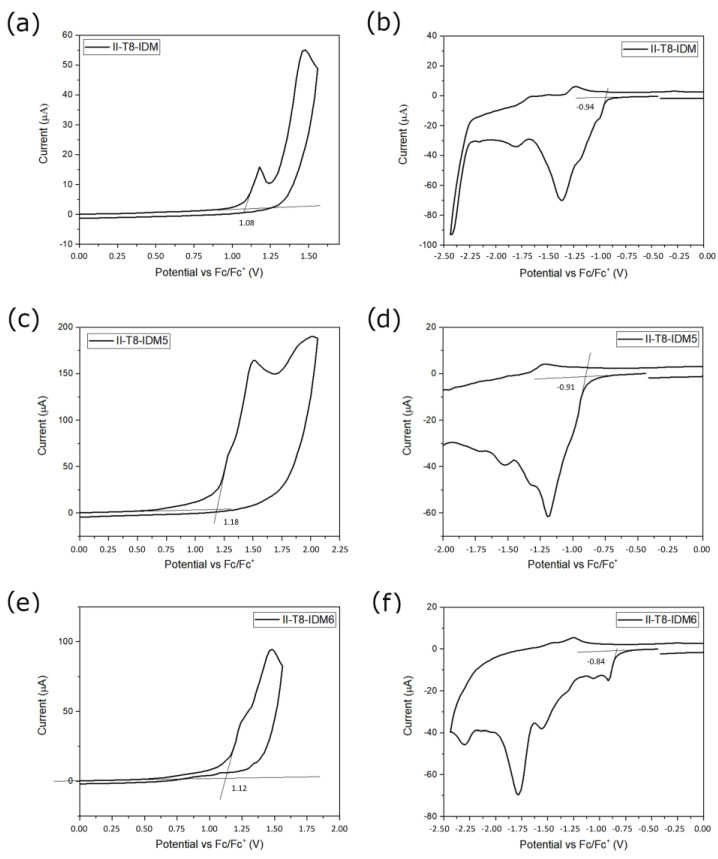
Cyclic voltammetry graphs of the **II**-based OSCs drop cast as thin film: (**a**,**c**,**e**): oxidation scan for **II-T8-IDM**, **II-T8-IDM5** and **II-T8-IDM6**; (**b**,**d**,**f**): reduction scan for **II-T8-IDM**, **II-T8-IDM5** and **II-T8-IDM6**.

Cyclic voltammetry experiments were carried on the **II**-based derivatives. Electrochemical experiments were performed on the dyes drop cast as thin films on a glassy carbon working electrode from chloroform solution in a three-electrode setup (the details are described in the Experimental Section). The recorded voltammograms are shown in Figure 5. For each material, we recorded a first experiment applying initially positive potential to determine the oxidation potential and then a second scan (on a different film of the same material), moving at first toward negative potentials, to measure the reduction potential. This procedure was used because both oxidation and reduction, for all the investigated systems, are irreversible phenomena. Because of this irreversible nature, we report, in Table 5, the oxidation and reduction potentials as the voltage value corresponding to the onset of the electrochemical process (see also Figure 5). These potentials are reported relative to the ferrocene/ferrocenium couple so that it is possible to obtain the energy values of HOMO and LUMO by applying the following relationship [39]:

(1) $$E_{HOMO\left(LUMO\right)}\left(eV\right)=-\left(E_{ox\left(red\right)}+5.1\right)eV$$

In Equation (1), *E_ox(red)_* represents the onset of the oxidation (reduction) potential of the molecule corrected vs. the half-wave potential of the ferrocene/ferrocenium couple, measured in the same experimental conditions. The correction factor 5.1 represents the formal potential of the ferrocene/ferrocenium couple in the Fermi scale, assuming that its value is 0.40 V, vs. SCE in acetonitrile solution, with tetrabutylammonium hexafluorophosphate 0.1 M as the supporting electrolyte [39].

The investigated **II**-based OSCs are all characterized by extremely stable HOMO energy, around or lower than −6.2 eV. The calculated LUMO energies are very low as well, with values lower than −4.16 eV that suggest potential n-type behavior for all the synthesized organic semiconductors. By comparing the brominated derivatives, the position of the bromine atom affects the electrochemical behavior: the isomer **II-T8-IDM5** is characterized by a deeper HOMO level as compared to that of **II-T8-IDM6**. The latter features instead a significantly lower LUMO energy. As compared to **II-T8-IDM**, the other dyes show both deeper HOMO and LUMO energies. In Table 5, we also report the calculated HOMO and LUMO energies. A good accord between experimental and calculated values is observed in the case of HOMO, while calculated values for LUMO are instead higher. The trend of the calculated LUMO energies is consistent with the experimental results, and the absolute value differences should be associated with the fact that the calculation was performed on the molecules in chloroform solution, while CV experiments were performed on thin films.

### 2.5. OFET Fabrication and Electrical Characterization

**Figure 6:**
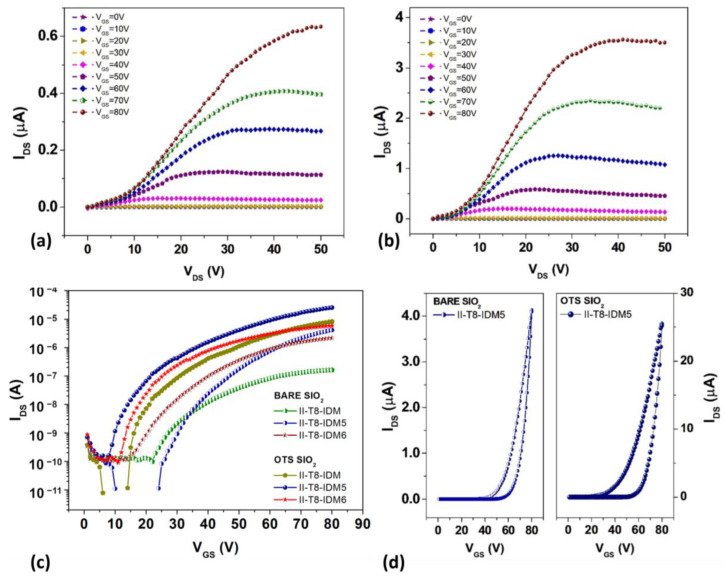
Output curves measured for **II-T8-IDM5** transistors fabricated on (**a**) bare and (**b**) OTS-treated SiO_2_ substrates. (**c**) Semi-log plot of a set of representative transfer curves measured in saturation regime for all the investigated compounds. (**d**) Transfer curves recorded in the saturation regime (V_DS_ = +50V) for **II-T8-IDM5** transistors fabricated on both bare and OTS-treated SiO_2_ substrates.

Thin-film field-effect transistors were fabricated from the molecules under investigation following the procedure described in the Experimental Section. After the spin-coating of the **II**-based OSCs, the transistors were annealed at 140 °C for 1 h under vacuum. The annealing temperature was selected after a careful investigation performed by XRD and UV–Vis analysis. Powder XRD spectra, shown in Figures S39–S41, are characterized by a strong reflection at low 2θ (around 4°). The increase in annealing temperature led to an enhancement in the intensity of this peak and to a slight shift toward higher angles, with the structure of the materials becoming more compact. This aspect was confirmed by UV–Vis analysis as well. In Figures S42–S44, indeed, the effect of annealing temperature on the optical response of the **II** derivative’s thin films clearly emerges: with this feature, the increase in the annealing temperature determines a red shift in the absorption up to 140 °C, which can be associated with a better structuration of the molecular layers. The OFETs were electrically tested using a well-established experimental protocol employed in previous studies dealing with similar **II**-based compounds [40]. In this regard, Figure 6 and Figure S45 provide a representative view of the observed electrical responses.

All the considered transistors were found to exhibit an n-type behavior, with the I_DS_ current being enhanced under the application of positive V_GS_ voltages. Conversely, no charge accumulation effect was observed for negative V_GS_ values. This occurrence confirms the pure electron-transporting character of these compounds, ascribable to the corresponding HOMO and LUMO energy levels. The output curves reported in Figure 6a,b and Figure S45a–d are characterized, in most cases, by a diode-like behavior of the I_DS_ current for the applied V_DS_ voltages smaller than 10 V. These electrical features are related to the impact of the contact resistance effects on the transistor response which, in general, tend to degrade the charge injection and extraction processes. In bottom-contact OFETs, this occurrence is mainly associated with the decreased quality of the film morphology near the thick pre-patterned gold electrodes [40].

Transfer curves in Figure 6 and Figure S45e,f confirm the electron accumulation phenomenon while showing with major clarity the occurrence of hysteresis effects, which, in general, produce a lowering of the I_DS_ current during the measurement (i.e., the I_DS_ current acquired in the forward is larger than that recorded in backward scan) because of the action of charge trapping mechanisms.

In Figure 6, a set of representative transfer curves measured for the various transistor types are reported in a semi-log plot. These measurements suggest that, for any compound, the devices based on an octadecyltrichlorosilane (OTS)-treated SiO_2_ surface display improved performance in terms of maximum currents, with the tendency to shift the onset voltages V_on_ (i.e., V_GS_ value where I_DS_ start increasing very rapidly) toward lower positive values. Both these observations are compatible with a reduced density of water-related charge trapping centers on the dielectric surface due to the enhanced hydrophobic nature of the OTS self-assembled monolayer.

**Figure 7:**
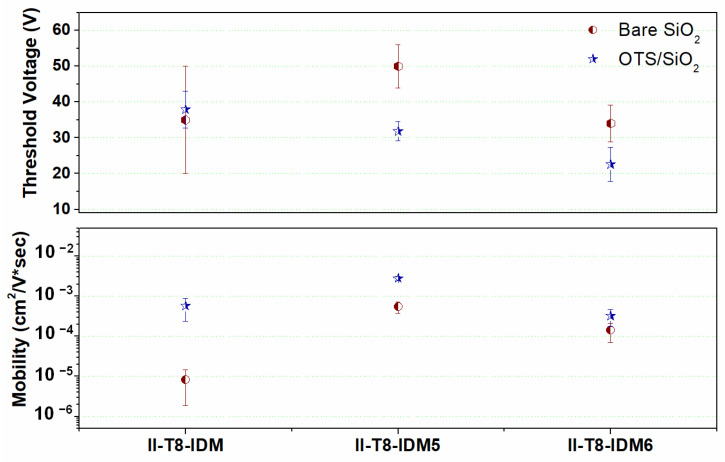
Threshold voltage (top panel) and mobility (bottom panel) average values extracted for **II-T8-IDM**-, **II-T8-IDM5**-, and **II-T8-IDM6**-based transistors fabricated on both bare and OTS-treated SiO_2_ substrates. Error bars represent the standard deviations.

Average mobility (µ) and threshold voltage (Vth) values, estimated from the analysis of the transfer curves achieved with the different molecules, are shown in Figure 7. The quantitative comparison among the µ values indicates that, both for devices on bare and OTS-functionalized SiO_2_ surfaces, the **II-T8-IDM5** compound exhibits the best performance. For this molecule, the average µ values were 2.8 × 10^−3^ and 5.5 × 10^−4^ cm^2^/V∙s, respectively, on treated and untreated SiO_2_. On OTS-functionalized surfaces, moreover, we found comparable average µ values for **II-T8-IDM** (µ_av_ = 5.7 × 10^−4^ cm^2^/V∙s) and **II-T8-IDM6** (µ_av_ = 3.3 × 10^−4^ cm^2^/V∙s).

Significantly, **II-T8-IDM** was the compound demonstrating by far the largest sensitivity on SiO_2_ surface, since an almost two orders of magnitude enhancement of average µ values induced by the OTS treatment was observed.

Overall, it should be mentioned that the mobility performance of the compound **II-T8-IDM5** overcomes those of previously investigated **II** derivatives bearing **IDM** terminal units [35]. The significantly superior electrical behavior of **II-T8-IDM5**, particularly when compared to that of its structural isomer **II-T8-IDM6**, is probably associated with its crystalline arrangement. A recent work investigating the behavior of two ITIC derivatives, end capped with a monochlorinated **IDM** acceptor group bearing chlorine substituents in position 5 or 6 of the indandione ring, demonstrated that the different positions of the chlorine atoms significantly affect the molecular packing [41]; in particular, the derivatives with the chlorine atoms in position 5 showed a better molecular planarity, a closer π–π interaction distance, and a 3D interpenetrating network structure that could be beneficial for electronic transport. Although we could not prepare single crystals of our **II** derivatives suitable for XRD analysis, based on the previous considerations, we suggest that the superior electrical performance of **II-T8-IDM5** could be provided by similar physical mechanisms.

## 3. Materials and Methods

### 3.1. General Information

All reagents were purchased from Fluorochem (Hadfield, UK) or Merck (Darmstad, Germany) and were used without any further purification. Compound **1** was prepared according to a previously reported procedure [36]. The chemical identity of all the precursors and final compounds was confirmed by 1H and 13C NMR analysis using Bruker 400 MHz Avance NMR spectrometers (Billerica, MA, USA). Positive and negative Reflectron MALDI spectra were recorded on an AB Sciex TOF/TOF 5800 instrument (Framingham, MA, USA) using 2,5-dihydroxybenzoic acid as the matrix. Melting points and solid–solid transition points were determined by temperature-controlled optical microscopy (Zeiss Axioskop (Oberkochen, Germany) polarizing microscope equipped with a Mettler Toledo HS82 (Columbus, OH, USA) hot stage) and DSC analysis performed on a Hitachi NEXTA DSC 200 instrument (Tokyo, Japan) under a nitrogen atmosphere at a scan rate of 10 °C/min. Absorption spectra were recorded on a JASCO V-750 UV–Vis spectrophotometer (Tokyo, Japan) at room temperature with a scanning rate of 200 nm/min; the experiments were carried out both in solution and as thin films deposed by spin-coating on glass substrates by means of a Laurell WS-650Mz-23NPP spin processor (Lansdale, PA, USA). Thin films of the final II-based OSCs were deposited by spin-coating on microscope coverslips and analyzed by wide-angle X-ray diffraction analysis using a Malvern Panalytical Empyrean multipurpose diffractometer (Malvern, UK), employing Cu Kα radiation (λ = 1.5418 Å) by a continuous scan of the diffraction angle 2θ in the interval 2–40° at a speed of 0.05252 °/s. Selected crystals of **IDM-6** were mounted on a Bruker-Nonius KappaCCD diffractometer (graphite monochromated MoKα radiation, λ = 0.71073 Å, CCD rotation images, thick slices, φ and ω scans to fill the asymmetric unit). Reduction of data and semiempirical absorption correction were performed using the SADABS program. The structures were solved by direct methods (SIR97 program) [42] and refined by the full-matrix least-squares method on F2 using the SHELXL-2016 program [43] with the aid of the Olex2 program [44]. H atoms bonded to C were generated stereochemically and refined via the riding model. For all H atoms, U_iso_(H) equal to 1.2 U_eq_ of the carrier atom was used. Crystal data and structure refinement details are reported in Table S1. The figures were generated using Mercury CSD [45]. Crystal data were deposited at the Cambridge Crystallographic Data Center, with assigned number CCDC 2470625. These data can be obtained free of charge from www.ccdc.cam.ac.uk/data_request/cif (accessed on 5 September 2025). FTIR spectra of the dyes were recorded on KBr pellets using a JASCO FTIR 4700 spectrometer. Cyclic voltammetry experiments were conducted using a conventional three-electrode setup made of an Ag/AgCl reference electrode, a Pt-spiral as a counter-electrode, and a glassy carbon-based working electrode; Palm Sens 4 (Houten, The Netherlands) was the potentiostat used, while all the electrodes were purchased from Basi (West Lafayette, IN, USA). The experiments were conducted on thin films of the **II**-based OSCs cast on the glassy carbon working electrode from 1 mg/mL chloroform solutions. For each scan (oxidation and reduction), a new film was deposed on the working electrode. Acetonitrile and t-butylammonium-hexafluorophosphate were used as the solvent and supporting electrolyte, respectively. Scan speed was fixed at 50 mV/s, and a positive (negative) direction was imposed for the oxidation (reduction) scan. Due to the poor reversibility of electrochemical oxidation (e.g., the film is detached from the electrode), only the first cycle of each scan is reported.

### 3.2. Synthesis

#### 3.2.1. Synthesis of (E)-1,1′-Bis(2-Octyldodecyl)-6,6′-Bis(4,4,5,5-Tetramethyl-1,3,2-Dioxaborolan-2-Yl)-[3,3′-Biindolinylidene]-2,2′-Dione (**II-B**)

A total of 1.42 g (1.45 mmol) of compound **1**, 0.816 g (3.21 mmol) of bis(pinacolato)diboron, and 0.837 g (8.53 mmol) of potassium acetate were added to 15 mL of dry dioxane in a two-neck round-bottom flask. The mixture was degassed by bubbling nitrogen through a needle for approximately 10 min. Then, 0.045 g (0.0615 mmol) of [1,1′-Bis(diphenylphosphino)-ferrocene]dichloropalladium(**II**) (PdCl_2_(dppf)) was added, and the temperature was increased to 80 °C. The system was kept under stirring at this temperature (under a nitrogen atmosphere) overnight. Then, the mixture was cooled and extracted with chloroform and brine in a 1:1 ratio. The organic phase was treated with anhydrous sodium sulphate, and the solvent was removed by means of a rotary evaporator. Finally, the solid residue was washed with 50 mL of cold methanol and filtered. Yield: 94%.

^1^H-NMR (CDCl_3_, 400 MHz), δ (ppm): 0.87 (m, 12 H); 1.24–1.27 (m, 64 H); 1.36 (s, 24 H); 1.95 (s broad, 2H); 3.69 (d, 4H, *J* = 7.5 Hz), 7.15 (s, 2H, *J* = 4.0 Hz); 7.47 (d, 2H, *J* = 8.0 Hz); 9.14 (d, 2H, *J* = 8.0 Hz).

^13^C-NMR (100 MHz, CDCl_3_): δ (ppm): 14.1, 22.7, 24.9, 26.4, 29.3, 29.6, 30.0, 31.6, 31.9, 36.1, 44.4, 67.1, 84.0, 113.5, 124.1, 128.7, 128.9,134.3, 144.4, 168.1.

MS (MALDI-TOF) *m/z*: calculated for C_68_H_113_B_2_N_2_O_6_ [(M + H)]^+^: 1075.88; found 1075.87.

#### 3.2.2. Synthesis of 5-Bromo-3-Octylthiophene-2-Carbaldehyde (**T8-CHO**)

A total of 4.60 g (16.7 mmol) of 2-bromo-3-octylthiophene was dissolved in 100 mL of dry tetrahydrofuran (THF), cooled to −78 °C, and kept under stirring under a nitrogen environment. After about 10 min, 10 mL of a 2 M solution of lithium diisopropylamide (LDA) in THF (20.0 mmol) was added. Then, after an additional 2 h, 2.3 mL of dry dimethylformamide (29.5 mmol) was added to the reaction mixture. The temperature was allowed to naturally rise to room value, and the system was kept under nitrogen overnight. The next day, the mixture was poured into 150 mL of water and extracted twice with petroleum ether (2 × 200 mL). The organic phase was treated with anhydrous sodium sulphate, and the solvent was removed. Purification of the reaction product was achieved by column chromatography using a 3:1 mixture of petroleum ether and diethyl ether. Yield: 86%.

^1^H-NMR (CDCl_3_, 400 MHz), δ (ppm): 0.88 (t, 3 H, *J* = 7.0 Hz); 1.28–1.32 (m, 4 H); 1.57–1.62 (m, 8 H); 2.57 (t, 2H, *J* = 7.5 Hz), 7.45 (s, H); 9.75 (s, H).

^13^C-NMR (100 MHz, CDCl_3_): δ (ppm): 14.4, 22.9, 29.4, 29.5, 29.6, 29.8, 32.1, 122.4, 137.0, 143.2, 144.3, 182.1.

#### 3.2.3. Synthesis of (E)-5,5′-(1,1′-Bis(2-Octyldodecyl)-2,2′-Dioxo-[3,3′-Biindolinylidene]-6,6′-Diyl)bis(4-Octylthiophene-2-Carbaldehyde) (**II-T8-CHO**)

A total of 2.20 g (2.10 mmol) of **II-B** and 1.59 g (2.10 mmol) of **T8-CHO** were dissolved in dry THF (35 mL) in a round-bottom flask under a nitrogen atmosphere. The reaction mixture was degassed by purging with a needle for 5 min. Following this, 5.4 mL of a 2.0 M potassium phosphate (K_3_PO_4_) solution was added to the flask. Next, 60 mg (0.052 mmol) of tetrakis(triphenylphosphine)-palladium [Pd(PPh_3_)_4_] was introduced into the reaction mixture; the system was taken to reflux and kept under stirring in a nitrogen environment overnight. After the reaction was completed, the solution was allowed to cool to room temperature. The crude reaction mixture was then extracted with a mixture of brine and chloroform (1:1). This procedure was repeated two additional times. Subsequently, the organic phase was dried with anhydrous sodium sulphate, and the solvent was removed using a rotary evaporator. The reaction solid was recrystallized twice with hot ethanol. The pure product was then isolated by recrystallization with dichloromethane and methanol. Yield: 33%.

^1^H-NMR (CDCl_3_, 500 MHz), δ (ppm): 0.83–0.89 (m, 18 H); 1.24–1.27 (m, 64 H); 1.33–1.36 (m, 24 H); 1.93 (s broad, 2H); 2.75 (t, 4H, *J* = 9.5 Hz), 3.71 (d, 4H, *J* = 7.5 Hz), 6.68 (s, 2H), 7.16 (d, 2H, *J* = 9.0 Hz), 7.68 (s, 2H); 9.27 (d, 2H, *J* = 8.5 Hz); 9.89 (s, 2H).

^13^C-NMR (100 MHz, CDCl_3_), δ (ppm): 14.1, 22.7, 26.5, 29.0, 29.1, 29.2, 29.3, 29.3, 29.4, 29.5, 29.5, 29.6, 30.0, 30.8, 31.7, 31.8, 36.4, 44.8, 108.6, 121.9, 122.9, 130.2, 133.0, 136.7, 137.5, 138.6, 141.2, 141.7, 145.7, 147.7, 168.3, 182.8.

FTIR (KBr pellet, cm^−1^): 802, 1097, 1261, 1350, 1461, 1610 (ν C=C), 1668 (ν C=O), 2852 (ν C-H), 2923 (ν C-H), 3453.

MS (MALDI-TOF) *m/z*: calculated for C_82_H_127_N_2_O_4_S_2_ [(M + H)^+^]: 1267.92; found 1268.02.

#### 3.2.4. Synthesis of 5-Bromo-1H-Indene-1,3(2H)-Dione and 6-Bromo-1H-Indene-1,3(2H)-Dione (Compound **2**)

4-bromophthalic anhydride (3.0 g, 13.00 mmol) was added to a 50 mL two-necked round bottom flask under a nitrogen atmosphere, and then 19 mL of acetic anhydride and 10 mL of triethylamine were successively added. Then, t-butyl acetoacetate (2.57 g, 16.25 mmol) was slowly added to the reaction mixture and kept under stirring at 40 °C overnight. The following day, the mixture was poured into 38 mL of ice water and 20 mL of concentrated HCl (15 mL), and the system was heated to 55 °C for about 30 min. After cooling to room temperature, the precipitate was filtered and washed with ice water, obtaining a brown solid (compound **2**), which can be directly used for the next step. Yield: 92%.

^1^H-NMR (500 MHz, CDCl_3_), δ (ppm): 8.11 (d, 1H, *J* = 1.2 Hz), 7.94 (dd, 1H, *J* = 8.1 Hz, *J* = 1.7 Hz), 7.84 (d, 1H, *J* = 8.1 Hz), 3.25 (s, 2H).

#### 3.2.5. Synthesis of 2-(5-Bromo-3-Oxo-2,3-Dihydro-1H-Inden-1-Ylidene)Malononitrile (**IDM-5**) and 2-(6-Bromo-3-Oxo-2,3-Dihydro-1H-Inden-1-Ylidene)Malononitrile (**IDM-6**)

Compound **2** (2.5 g, 11.1 mmol) and malononitrile (2.0 g, 22.2 mmol) were mixed in 75 mL of ethanol. Then, anhydrous sodium acetate (1.9 g, 23.2 mmol) was added to the reaction, and the mixture was stirred at room temperature for 1 h. At the end of the reaction, the reaction mixture was poured into 75 mL of water, and 5 mL of concentrated HCl was added dropwise to acidify the mixture at pH = 2. After complete precipitation of the solid, the crude product was filtered and washed with water.

The resulting solid was dissolved in acetonitrile to afford a solution with a concentration of approximately 90 mg mL^−1^, and the resulting suspension was stirred at ambient temperature overnight. On the following day, the mixture was subjected to filtration, yielding solution enriched in **IDM-6** (2:1, as determined by 1H NMR analysis) and a solid residue enriched in **IDM-5** (2:1). The solvent from the **IDM-6**-rich solution was removed at reduced pressure, and the resulting solid was recrystallized from hot dichloroethane. In this way, a pure fraction of **IDM**6 was obtained (Yield: 25%). The **IDM-5**-rich solid residue from the first filtration was treated a second time with acetonitrile (90 mg/mL again) and kept under stirring at room temperature overnight. The system was then filtrated, and the solid fraction recrystallized by hot chloroform. **IDM-5** was so obtained as pure compound (final yield: 27%).

**IDM-6**

^1^H NMR (500 MHz, CDCl_3_), δ (ppm): 8.76 (s, 1H), 7.96 (d, 1H, *J* = 8.1 Hz, *J* = 1.7 Hz), 7.83 (d, 1H, *J* = 8.2 Hz), 3.72 (s, 2H).

^13^C NMR (100 MHz, CDCl_3_, δ (ppm): 43.2, 111.7, 125.7, 128.9, 131. 8, 138.9, 143.6, 164.6, 193.5.

**IDM-5**

^1^H NMR (500 MHz, CDCl_3_), δ (ppm): 8.49 (d, 1H, *J* = 8.6 Hz), 8.11 (d, 1H, *J* = 1.7 Hz), 7.98 (dd, 1H, *J* = 8.5 Hz, *J* = 1.8 Hz), 3.74 (s, 2H).

^13^C NMR (100 MHz, CDCl_3_), δ (ppm): 43.2, 111.9, 124.6, 126.8, 131.6, 132.9, 134.4, 137.5, 140.9, 141.7, 164.9, 193.4.

#### 3.2.6. Synthesis of 2,2′-((2Z,2′Z)-((((E)-1,1′-Bis(2-Octyldodecyl)-2,2′-Dioxo-[3,3′-Biindolinylidene]-6,6′-Diyl)Bis(4-Octylthiophene-5,2-Diyl))Bis(Methaneylylidene))Bis(3-Oxo-2,3-Dihydro-1H-Indene-2,1-Diylidene))Dimalononitrile (**II-T8-IDM**)

A total of 220 mg (0.177 mmol) of **II-T8-CHO**, 114 mg (0.721 mmol) of 1,1-dicyanomethylene-3-indanone, and 50 mg (0.561 mmol) of β-alanine were added to a two-neck round-bottom flask and placed under a nitrogen atmosphere. 16.0 mL of anhydrous dichloroethane and 4.0 mL of ethanol were added to the reaction system. The reaction was kept under stirring at reflux overnight. When the reaction concluded, the mixture was extracted with chloroform and brine in a 1:1 ratio. The organic phase was treated with anhydrous sodium sulphate, and the solvent was removed. The crude solid was dissolved in chloroform (20 mL) and precipitated in 100 mL of methanol; the obtained mixture was filtered, obtaining the pure product as a blue solid. Yield: 77%.

^1^H NMR (CDCl_3_, 500 MHz), δ (ppm): 0.82–0.90 (m, 18 H); 1.21–1.27 (m, 60 H); 1.37–1.40 (m, 24 H), 1.70 (q, 4H, *J* = 5.0 Hz), 1.98 (s, broad, 2H), 2.79 (t, 4H, *J* = 10.0 Hz), 3.76 (d, 4H, *J* = 7.4 Hz), 6.98 (s, 2H), 7.28 (s, 2H), 7.80 (m, 6H), 7.96 (d, 2H, *J* = 7.4 Hz), 8.74 (d, 2H, *J* = 7.6 Hz), 8.88 (s, 2H), 9.31 (d, 2H, *J* = 8.3 Hz).

^13^C NMR (100 MHz, CDCl_3_), δ (ppm): 14.1, 22.6, 26.6, 28.8, 29.4, 29.6, 30.1, 30.8, 31.7, 31.9, 44.8, 108.4, 114.3, 114.4, 122.3, 123.1, 123.4, 123.9, 125.4, 130.1, 133.1, 134.7, 135.3, 137.0, 141.9, 145.6, 147.1, 154.6, 160.5, 168.3, 188.0.

FTIR (KBr pellet, cm^−1^): 802, 1097, 1261, 1334, 1452, 1546, 1610 (ν C=C), 1693 (ν C=O), 2218 (ν C≡N), 2852(ν C-H), 2921 (ν C-H), 3434.

MS (MALDI-TOF) *m/z*: calculated for C_106_H_135_N_6_O_4_S_2_ [(M + H)^+^]: 1620.00; found 1620.09.

#### 3.2.7. Synthesis of 2,2′-((2Z,2′Z)-((((E)-1,1′-Bis(2-Octyldodecyl)-2,2′-Dioxo-[3,3′-Biindolinylidene]-6,6′-Diyl)Bis(4-Octylthiophene-5,2-Diyl))Bis(Methaneylylidene))Bis(-Bromo-3-Oxo-2,3-Dihydro-1H-Indene-2,1-Diylidene))Dimalononitrile (**II-T8-IDM5**)

A total of 100 mg (0.0789 mmol) of **II-T8-CHO**, 107 mg (0.394 mmol) of **IDM5**, and 28 mg (0.316 mmol) of β-alanine were added to a two-neck round-bottom flask and placed under a nitrogen atmosphere. A total of 12.0 mL of anhydrous DCE and 3.0 mL of ethanol were added to the reaction system. The reaction was kept under stirring at reflux overnight. The reaction mixture was cooled at room temperature, and the precipitation of a solid occurred. The precipitate was recovered by filtration, redissolved in 20 mL of chloroform, and precipitated in 100 mL of methanol. A deep blue solid was recovered by suction filtration. Yield: 64%.

^1^H NMR (CDCl3, 500 MHz), δ (ppm): 0.82–0.90 (m, 18 H); 1.21–1.26 (m, 60 H); 1.38–1.43 (m, 24 H), 1.70 (t, 4H, *J* = 8.0 Hz), 1.98 (s, broad, 2H), 2.79 (t, 4H, *J*= 8.0 Hz), 3.75 (d, 4H, *J* = 7.8 Hz), 6.98 (s, 2H), 7.29 (s, 2H), 7.81 (m, 2H), 7.90 (dd, 2H, *J* = 7.4 Hz, *J* = 1.8 Hz), 8.06 (d, 2H, *J* = 1.07 Hz), 8.59 (d, 2H, *J* = 8.5 Hz), 8.89 (s, 2H), 9.31 (d, 2H, *J* = 8.3 Hz).

^13^C NMR (100 MHz, CDCl_3_), δ (ppm): 14.1, 22.6, 26.6, 28.8, 29.3, 29.6, 30.0, 30.8, 31.7, 31.9, 44,8, 108.2, 112.8, 113.5, 123.0, 123.1, 125.1, 128.2, 129.9, 130.2, 133.0, 135.3, 137.0, 138.1, 141.0, 142.0, 145.2, 147.2, 155.1, 159.1, 168.1, 187.3.

FTIR (KBr pellet, cm^−1^): 802, 1099, 1263, 1309, 1381, 1545, 1608 (ν C=C), 1693 (ν C=O), 2220 (ν C≡N), 2850 (ν C-H), 2922 (ν C-H), 3438.

MS (MALDI-TOF) *m/z*: Calculated for C_106_H_132_Br_2_N_6_O_4_S_2_Na: 1797.80[(M + Na)^+^]; 1799.80[((M + 2) + Na)^+^]; 1801.80 [((M + 4) + Na)^+^]; found 1798.04 [(M + Na)^+^]; 1800.00 [((M + 2) + Na)^+^] and 1802.02 [((M + 4) + Na)^+^]

#### 3.2.8. Synthesis of 2,2′-((2Z,2′Z)-((((E)-1,1′-Bis(2-Octyldodecyl)-2,2′-Dioxo-[3,3′-Biindolinylidene]-6,6′-Diyl)Bis(4-Octylthiophene-5,2-Diyl))Bis(Methaneylylidene))Bis(6-Bromo-3-Oxo-2,3-Dihydro-1H-Indene-2,1-Diylidene))Dimalononitrile (**II-T8-IDM6**)

**II-T8-IDM6** was synthetized according to the same procedure adopted for **II-T8-IDM5**, the only exception being that **IDM6** was used instead of **IDM5**. Yield: 59%.

^1^H NMR (CDCl3, 500 MHz), δ (ppm): 0.82–0.90 (m, 18 H); 1.21–1.26 (m, 60 H); 1.37–1.39 (m, 24 H), 1.70 (t, 4H, *J* = 10.0 Hz), 1.96 (s, broad, 2H), 2.79 (t, 4H, *J* = 10.0 Hz), 3.75 (d, 4H, *J* = 7.2 Hz), 6.97 (s, 2H), 7.28 (s, 2H), 7.81 (m, 2H), 7.90 (dd, 2H, *J* = 8.0 Hz, *J* = 0.8 Hz), 8.89 (m, 4H), 9.31 (d, 2H, *J* = 8.1 Hz).

^13^C NMR (100 MHz, CDCl_3_), δ (ppm): 14.1, 22.6, 26.6, 29.3, 29.6, 30.1, 31.9, 44.8, 108.4, 114.1, 122.4, 122.8, 123.2, 125.0, 128.4, 130.3, 130.6, 133.1, 135.5, 135.8, 137.3, 137.8, 138.4, 141.3, 142.2, 145.7, 147.6, 155.5, 159.0, 168.3, 187.0.

FTIR (KBr pellet, cm^−1^): 802, 1018, 1097, 1261, 1315, 1382, 1544, 1612 (ν C=C), 1693 (ν C=O), 2218 (ν C≡N), 2852 (ν C-H), 2919 (ν C-H), 3428.

MS (MALDI-TOF) *m/z*: Calculated for C_106_H_133_Br_2_N_6_O_4_S_2_: 1775.82[(M + H)^+^]; 1777.82 [((M + 2) + H)^+^]; 1779.82 [((M + 4) + H)^+^]; found 1776.02 [(M + H)^+^]; 1777.99 [((M + 2) + H)^+^] and 1779.93 [((M + 4) + H)^+^].

### 3.3. DFT Analysis

All electronic computations were carried out at the density functional theory (DFT) level using the PBE0 functional, together with the polarized 6–31 + G(d,p) basis set [46]. That level of computations should provide reliable results, according to previous work [47]. Geometry optimizations and computations of normal coordinates and harmonic vibrational frequencies were carried out using the Gaussian package (G16) [48]. To verify that the identified stationary points were real minima on the potential energy surface, the calculation of the vibrational frequencies was carried out, and no imaginary values were found in any case. Time-dependent DFT (TDDFT) was employed for treating all excited states and to obtain the UV–Vis absorption spectra. The solvent (chloroform) effect has been included in the computation through the polarizable continuum model (PCM), as implemented in the Gaussian 16 code [49]. The long alkyl chains were replaced by methyl groups in all the electronic computations, as routinely implemented in DFT studies [50,51]

### 3.4. OFET Fabrication and Electrical Characterization

The electrical properties of the synthesized molecules were investigated through the fabrication of field-effect transistors. To this aim, the compounds were dissolved in 1,1-2,2-tetrachloroethane (5 mg/mL) and deposited as thin films by spin-coating at 1000 rpm for 1 min and then at 2000 rpm for 30 s on n-type highly doped (resistivity = 3 × 10^−2^ ohm∙cm) silicon substrates (thickness of 500 µm), working as gate contacts and covered with 200 nm thick silicon dioxide (SiO_2_) insulating barriers equipped, in turn, with interdigitated gold electrodes as drain and source contacts. This bottom-contact bottom-gate configuration has been widely utilized recently to reliably characterize a wide number of organic semiconductors displaying both unipolar and ambipolar behavior [52]. In this study, we considered devices achieved by using both untreated (bare) and octadecyltrichlorosilane (OTS)-functionalized SiO_2_ surfaces. The functionalization with OTS was carried out in several steps: the bare substrates were activated by placing them in piranha solution for about 1 min; the substrates were then abundantly rinsed with distilled water, and then sonicated in methanol (5 min) and isopropanol (5 min). The washed substrates were finally dried with nitrogen and placed (under a nitrogen environment) in a flask containing 50 mL of toluene. Storing the system under an inert environment, 0.1 mL of OTS were added. The system was kept at room temperature overnight, and then the substrates were recovered, washed abundantly with fresh toluene, and dried at 80 °C under vacuum for 1 h. After their fabrication, the devices were electrically tested using the same protocol adopted for similar compounds [35]. Namely, the devices were mounted in the vacuum chamber of a Janis cryogenic probe station which was evacuated down to about 10^−5^ mbar. Then, both transfer (*I_ds_* drain–source current measured as a function of the *V_GS_* gate-source voltage, while maintaining fixed *V_DS_* drain–source voltage) and output (*I_DS_* vs. *V_DS_* at fixed *V_GS_*) curves were acquired by using a Keithley 4200 Semiconducting Parameter Analyzer (Solon, OH, USA) connected to the probe station terminals through triaxle cables. Output curves were recorded by sweeping *V_DS_* from 0 to 50 V for different *V_GS_* values, from 0 to 80 V, with a step of 10 V. Transfer curves were measured in a saturation regime by setting the *V_DS_* at 50 V and the scanning *V_GS_* from 0 and 80 V and backward, with the purpose of highlighting the possible occurrence of hysteresis phenomena. Transfer curves were then processed to estimate mobility (µ) and threshold voltage (*V_th_*) values, based on the MOSFET equation valid in the saturation regime:

(2) $$\sqrt{I_{ds}}=\sqrt{\frac{W}{2L}C_{ox}\mu *}(V_{GS}-V_{th})$$

which was used to linearly fit the square root of the experimental curve. In this expression, *C_ox_* (unit area capacitance of the insulating SiO_2_ barrier) and *W*/*L* (ratio between width and length of the active channel) were, respectively, 17.25 nF/cm^2^ and 550.

## 4. Conclusions

Three new **II**-based organic semiconductors were synthesized and characterized in this work. The new molecules represent a structural evolution of a previously reported material [35], based on an isoindigo electron acceptor core, symmetrically linked to electron donor thiophene rings and end capped with 1-Dicyanomethylene-3-Indanone (**IDM**). The structural variations implemented in this prototypal molecular structure were the insertion of an octyl chain on the thiophene rings (**II-T8-IDM)** and further, the insertion of a bromine atom in position 5 or 6 of the **IDM** ring (respectively, **II-T8-IDM5** and **II-T8-IDM6** compounds). The functionalization of thiophene rings with the alkyl chain displayed the effect of increasing the solubility of the new molecules as compared to that of the original; this effect was also accompanied by a significant reduction in the melting point. The three new molecules were optically characterized, both in solution and as thin film. In regards to the experiments conducted in solution, by comparing the not-brominated **II-T8-IDM** with the other two compounds, we can observe a slight redshift (about 10 nm) of the optical absorption maxima, regardless of the position of the bromine atom. In the case of thin film measurements, the same red-shift is observed, but the position of the bromine atom appears to play a role in the optical bandgap (graphically determined by the endset of the absorption): specifically, **II-T8-IDM6** features a bandgap of 1.55 eV, significantly lower than those of its constitutional isomer **II-T8-IDM5** (1.64 nm) and of **II-T8-IDM** (1.66 nm). The electronic properties of the new **II**-based OSCs were studied at the DFT level; qualitatively, the calculated optical spectra well-reproduce the experimental results. The optimized geometry shows that the molecules present a significant distortion from planarity around the bond linking the thiophene ring to the **II** core, caused by the steric hindrance provided by the octyl chain. For all the molecules, electron density distribution moves (although slightly) from the central part of the molecule towards the peripheral acceptor group upon excitation from HOMO to LUMO. The new molecules were also characterized (as thin films) in regards to their electrochemical properties; from the onset of oxidation and the reduction potentials, it was possible to estimate the LUMO and HOMO energies. All the molecules present LUMO energies well below −4.0 eV, so that the n-type charge transport behavior can be reasonably expected. The bromination of the terminal **IDM** groups had the effect of stabilizing LUMO energies, and this effect was more significant when it occurred in position 6. All the **II**-based OSCs were used for the fabrication of OFET devices, and their charge transport behavior and mobility were investigated. All the dyes are unipolar, n-type semiconductors, with the mobility ranging between 10^4^ and 10^−3^ cm^2^/V∙s, in line with the values typical reported for the most-used NFAs [24]. **II-T8-IDM5** shows the best mobility value, one order of magnitude higher than that of its isomer **II-T8-IDM6**; these results are consistent with a previous paper [41], in which the bromine substitution on position 5 of the terminal **IDM** group led to better electrical properties. The different position of the bromine atoms in the two isomers probably influences the crystalline arrangement and hence, the electron transport. Interestingly, the bromination in position 5 of the terminal acceptor doubled the electron mobility as compared to the that of the previously reported parent compound **II-T-IDM** [35], notwithstanding the introduction on the thiophene rings of a further alkyl chain that, although necessary to increase solubility, unavoidably led to an increased disorder in the molecular packing. In conclusion, the electrical and optical properties of the new **II** derivatives here reported make these molecules interesting candidates as non-fullerene acceptors in bulk heterojunction solar cells, and we plan to test their photovoltaic performance in forthcoming works.

## Figures and Tables

**Table 1:** Optical properties of the **II**-based compounds in chloroform solution.

Dyes	λ_1_/ε_1_^a^	λ_2_/ε_2_^a^	λ_3_/ε_3_^a^	λ_4_/ε^a^	λ_5_/ε_5_^a^
**II-T8-IDM**	577/4.7 × 10^4^	506/4.9 × 10^4^	333/2.8 × 10^4^	292/4.8 × 10^4^	242/3.50 × 10^4^
**II-T8-IDM5**	587/3.9 × 10^4^	512/3.8 × 10^4^	338/3.4 × 10^4^	293/3.5 × 10^4^	242/3.53 × 10^4^
**II-T8-IDM6**	587/4.9 × 10^4^	515/4.6 × 10^4^	-	295/6.8 × 10^4^	241/4.70 × 10^4^

Absorption maxima wavelengths are expressed in nm, while the molar extinction coefficient is expressed in dm^3^∙mol^−1^∙cm^−1^.

**Table 2:** Optical properties of the **II**-based OSCs as thin film ^a^.

Dyes	λ (nm)	E_g_ (eV) ^b^
**II-T8-IDM**	650/552(sh)	1.66
**II-T8-IDM5**	660/553(sh)	1.64
**II-T8-IDM6**	657/555(sh)/722(sh)	1.55

^a^ Thin films prepared by spin-coating from 1,1-2,2-tetrachloroethane solution and then annealed for 1 h at 140 °C; ^b^ calculated graphically (see Figures S30–S32) from the endset of the absorption (E_g_ = 1240/λ_endset_).

**Table 3:** Calculated dihedral angles (degrees), as defined in the figure above, in the optimized ground state geometry of the **II** derivatives.

Dyes	D1 (°)	D1′ (°)	D2 (°)	D2′ (°)	D3 (°)
**II-T8-IDM**	−10.10	−13.77	37.41	−38.86	12.78
**II-T8-IDM5**	−8.29	−11.10	37.44	−38.97	14.77
**II-T8-IDM6**	−10.49	−10.49	37.62	−38.96	14.89

**Table 4:** Computed electronic and optical properties of the synthesized **II** derivatives in chloroform.

	λ_abs_ (nm)	F ^a^	μ_GS_ (D) ^b^	μ_ES_ (D) ^c^	E_g_ (eV) ^d^	E_g_^exp^ (eV) ^e^
**II-T8-IDM**	637	1.97	6.07	4.95	1.95	1.80
**II-T8-IDM5**	642	2.06	6.82	5.72	1.93	1.78
**II-T8-IDM6**	644	2.06	8.85	7.35	1.93	1.77

^a^ Oscillator strength; ^b^ dipole moment of the ground state; ^c^ dipole moment of the first excited state; ^d^ calculated optical bandgap; ^e^ experimental optical bandgap graphically determined from the optical spectra in chloroform solutions.

**Table 5:** Electrochemical properties of the studied **II** derivatives as thin films.

**Dyes**	**E_ox_ (V) ^a^**	**HOMO (eV) ^b^**	**HOMO (eV) ^c^**	**E_red_ (V) ^a^**	**LUMO (eV) ^b^**	**LUMO (eV) ^c^**
**II-T8-IDM**	1.08	−6.18	−6.14	−0.94	−4.16	−3.63
**II-T8-IDM5**	1.18	−6.28	−6.18	−0.91	−4.19	−3.69
**II-T8-IDM6**	1.12	−6.22	−6.18	−0.84	−4.26	−3.70

^a^ Oxidation and reduction potential are measured vs. ferrocene/ferrocenium; ^b^ HOMO and LUMO energies are calculated as E_HOMO(LUMO) (eV) = −(E_ox(red)_ +5.1) eV, according to reference [39] ^c^ calculated energy values of HOMO and LUMO in chloroform solution.

## Data Availability

Data are contained within the article or Supplementary Material.

## Supplementary Materials

The following supporting information can be downloaded at: https://www.mdpi.com/article/10.3390/molecules30183672/s1: Ortep diagram of compound **IDM-6** (Figure S1); 1H NMR, 13C NMR, Maldi-TOF MS, and FTIR spectra for all the precursors and the final dyes (Figures S2–S26); DSC for **II-T8-IDM, II-T8-IDM5**, and **II-T8-IDM6** molecules (Figures S27–S29); Tauc plot for **II-T8-IDM, II-T8-IDM5**, and **II-T8-IDM6** molecules (Figures S30–S32); Computed optimized molecular geometry and optical spectra for **II-T8-IDM, II-T8-IDM5**, and **II-T8-IDM6** molecules (Figures S33–S38); XRD diffraction spectra of thin films of the compound **II-T8-IDM**, **II-T8_IDM5** and **II-T8_IDM-6** compounds (Figures S39–S41); Optical spectra of thin films of the compounds **II-T8-IDM**, **II-T8_IDM5** and **II-T8_IDM-6** annealed for 1 h at different temperatures (Figures S42–S44); Selected output and transfer curves for **II-T8-IDM**- and **II-T8-IDM6**-based transistors (Figure S45); Crystal data and structure refinement details for **IDM-6** (Table S1); Thermal properties for **II-T8-IDM, II-T8-IDM5**, and **II-T8-IDM6** molecules (Table S2); Main optical transitions (computed) for **II-T8-IDM, II-T8-IDM5**, and **II-T8-IDM6** molecules (Table S3).

## References

[1] Facchetti A. (2007). Semiconductors for organic transistors. Mater. Today.

[2] Zhang Q., Hu W., Sirringhaus H., Müllen K. (2022). Recent Progress in Emerging Organic Semiconductors. Adv. Mater..

[3] Forrest S.R. (2020). Organic Electronics: Foundations to Applications.

[4] Basiricò L., Mattana G., Mas-Torrent M. (2022). Editorial: Organic Electronics: Future Trends in Materials, Fabrication Techniques and Applications. Front. Phys..

[5] Root S.E., Savagatrup S., Printz A.D., Rodriquez D., Lipomi D.J. (2017). Mechanical Properties of Organic Semiconductors for Stretchable, Highly Flexible, and Mechanically Robust Electronics. Chem. Rev..

[6] Xu X., Zhao Y., Liu Y. (2023). Wearable Electronics Based on Stretchable Organic Semiconductors. Small.

[7] O’Connor T.F., Zaretski A.V., Savagatrup S., Printz A.D., Wilkes C.D., Diaz M.I., Sawyer E.J., Lipomi D.J. (2016). Wearable organic solar cells with high cyclic bending stability: Materials selection criteria. Sol. Energy Mater. Sol. Cells.

[8] Yang A., Yan F. (2021). Flexible Electrochemical Biosensors for Health Monitoring. ACS Appl. Electron. Mater..

[9] Hong G., Gan X., Leonhardt C., Zhang Z., Seibert J., Busch J.M., Bräse S. (2021). A Brief History of OLEDs—Emitter Development and Industry Milestones. Adv. Mater..

[10] Kubo T., Häusermann R., Tsurumi J., Soeda J., Okada Y., Yamashita Y., Akamatsu N., Shishido A., Mitsui C., Okamoto T. (2016). Suppressing molecular vibrations in organic semiconductors by inducing strain. Nat. Commun..

[11] Paulsen B.D., Tybrandt K., Stavrinidou E., Rivnay J. (2020). Organic mixed ionic–electronic conductors. Nat. Mater..

[12] Landi A., Reisjalali M., Elliott J.D., Matta M., Carbone P., Troisi A. (2023). Simulation of polymeric mixed ionic and electronic conductors with a combined classical and quantum mechanical model. J. Mater. Chem. C.

[13] Liu K., Ouyang B., Guo X., Guo Y., Liu Y. (2022). Advances in flexible organic field-effect transistors and their applications for flexible electronics. npj Flex. Electron..

[14] Schwartz G., Tee B.C.-K., Mei J., Appleton A.L., Kim D.H., Wang H., Bao Z. (2013). Flexible polymer transistors with high pressure sensitivity for application in electronic skin and health monitoring. Nat. Commun..

[15] Quinn J.T.E., Zhu J., Li X., Wang J., Li Y. (2017). Recent progress in the development of n-type organic semiconductors for organic field effect transistors. J. Mater. Chem. C.

[16] Meng B., Liu J., Wang L. (2021). Recent development of n-type thermoelectric materials based on conjugated polymers. Nano Mater. Sci..

[17] Gao X., Hu Y. (2014). Development of n-type organic semiconductors for thin film transistors: A viewpoint of molecular design. J. Mater. Chem. C.

[18] Liu Y. Advantages of CMOS Technology in Very Large Scale Integrated Circuits. Proceedings of the AIEE‘21: Proceedings of the 2021 2nd International Conference on Artificial Intelligence in Electronics Engineering.

[19] Xie Y., Ding C., Jin Q., Zheng L., Xu Y., Xiao H., Cheng M., Zhang Y., Yang G., Li M. (2024). Organic transistor-based integrated circuits for future smart life. SmartMat.

[20] Yeh N., Yeh P. (2013). Organic solar cells: Their developments and potentials. Renew. Sustain. Energy Rev..

[21] Liu W., Xu X., Yuan J., Leclerc M., Zou Y., Li Y. (2021). Low-Bandgap Non-fullerene Acceptors Enabling High-Performance Organic Solar Cells. ACS Energy Lett..

[22] Kim M., Ryu S.U., Park S.A., Pu Y.-J., Park T. (2021). Designs and understanding of small molecule-based non-fullerene acceptors for realizing commercially viable organic photovoltaics. Chem. Sci..

[23] Li S., Zhang H., Yue S., Yu X., Zhou H. (2021). Recent advances in non-fullerene organic photovoltaics enabled by green solvent processing. Nanotechnology.

[24] Zhang G., Zhao J., Chow P.C.Y., Jiang K., Zhang J., Zhu Z., Zhang J., Huang F., Yan H. (2018). Nonfullerene Acceptor Molecules for Bulk Heterojunction Organic Solar Cells. Chem. Rev..

[25] Chen H., Huang Y., Zhang R., Mou H., Ding J., Zhou J., Wang Z., Li H., Chen W., Zhu J. (2025). Organic solar cells with 20.82% efficiency and high tolerance of active layer thickness through crystallization sequence manipulation. Nat. Mater..

[26] Feng K., Wang G., Lian Q., Gámez-Valenzuela S., Li B., Ding R., Yang W., Wang K., Zeng J., Zhang Y. (2025). Non-fullerene electron-transporting materials for high-performance and stable perovskite solar cells. Nat. Mater..

[27] Hu Q., Chen W., Yang W., Li Y., Zhou Y., Larson B.W., Johnson J.C., Lu Y.-H., Zhong W., Xu J. (2020). Improving Efficiency and Stability of Perovskite Solar Cells Enabled by A Near-Infrared-Absorbing Moisture Barrier. Joule.

[28] Jiang J., Jin Z., Lei J., Wang Q., Zhang X., Zhang J., Gao F., Liu S. (2017). ITIC surface modification to achieve synergistic electron transport layer enhancement for planar-type perovskite solar cells with efficiency exceeding 20%. J. Mater. Chem. A.

[29] Forti G., Nitti A., Osw P., Bianchi G., Po R., Pasini D. (2020). Recent Advances in Non-Fullerene Acceptors of the IDIC/ITIC Families for Bulk-Heterojunction Organic Solar Cells. Int. J. Mol. Sci..

[30] Lin Y., Wang J., Zhang Z., Bai H., Li Y., Zhu D., Zhan X. (2015). An Electron Acceptor Challenging Fullerenes for Efficient Polymer Solar Cells. Adv. Mater..

[31] Yuan J., Zou Y. (2022). The history and development of Y6. Org. Electron..

[32] Ge J., Xie L., Peng R., Ge Z. (2023). Organic Photovoltaics Utilizing Small-Molecule Donors and Y-Series Nonfullerene Acceptors. Adv. Mater..

[33] Feng L., Yuan J., Zhang Z., Peng H., Zhang Z.-G., Xu S., Liu Y., Li Y., Zou Y. (2017). Thieno[3,2-*b*]pyrrolo-Fused Pentacyclic Benzotriazole-Based Acceptor for Efficient Organic Photovoltaics. ACS Appl. Mater. Interfaces.

[34] Luo D., Brabec C.J., Kyaw A.K.K. (2023). Non-fused ring electron acceptors for high-performance and low-cost organic solar cells: Structure-function, stability and synthesis complexity analysis. Nano Energy.

[35] Carella A., Franzini M., Fusco S., Centore R., Barra M., Chiarella F., Cassinese A., Bonomo M., Nejrotti S., Carbone M. (2022). Isoindigo dyes functionalized with terminal electron-withdrawing groups: Computational, optical and electrical characterization. Dye. Pigment..

[36] Maglione C., Carella A., Centore R., Fusco S., Velardo A., Peluso A., Colonna D., Di Carlo A. (2016). Tuning optical absorption in pyran derivatives for DSSC. J. Photochem. Photobiol. A Chem..

[37] Lu S., Li F., Zhang K., Zhu J., Cui W., Yang R., Yu L., Sun M. (2020). Halogenation on terminal groups of ITIC based electron acceptors as an effective strategy for efficient polymer solar cells. Sol. Energy.

[38] Landi A., Landi A., Velardo A., Peluso A. (2022). Efficient Charge Dissociation of Triplet Excitons in Bulk Heterojunction Solar Cells. ACS Appl. Energy Mater..

[39] Cardona C.M., Li W., Kaifer A.E., Stockdale D., Bazan G.C. (2011). Electrochemical Considerations for Determining Absolute Frontier Orbital Energy Levels of Conjugated Polymers for Solar Cell Applications. Adv. Mater..

[40] Chiarella F., Barra M., Carella A., Parlato L., Sarnelli E., Cassinese A. (2016). Contact-resistance effects in PDI8-CN 2 n-type thin-film transistors investigated by Kelvin-probe potentiometry. Org. Electron..

[41] Lai H., Chen H., Zhou J., Qu J., Chao P., Liu T., Chang X., Zheng N., Xie Z., He F. (2019). Isomer-free: Precise Positioning of Chlorine-Induced Interpenetrating Charge Transfer for Elevated Solar Conversion. iScience.

[42] Altomare A., Burla M.C., Camalli M., Cascarano G.L., Giacovazzo C., Guagliardi A., Moliterni A.G.G., Polidori G., Spagna R. (1999). SIR97: A new tool for crystal structure determination and refinement. J. Appl. Crystallogr..

[43] Sheldrick G.M. (2015). Crystal structure refinement with *SHELXL*. Acta Crystallogr. Sect. C Struct. Chem..

[44] Dolomanov O.V., Bourhis L.J., Gildea R.J., Howard J.A.K., Puschmann H. (2009). OLEX2: A complete structure solution, refinement and analysis program. J. Appl. Cryst..

[45] Macrae C.F., Bruno I.J., Chisholm J.A., Edgington P.R., McCabe P., Pidcock E., Rodriguez-Monge L., Taylor R., van de Streek J., Wood P.A. (2008). *Mercury CSD 2.0*—New features for the visualization and investigation of crystal structures. J. Appl. Crystallogr..

[46] Adamo C., Barone V. (1999). Toward reliable density functional methods without adjustable parameters: The PBE0 model. J. Chem. Phys..

[47] Carella A., Landi A., Bonomo M., Chiarella F., Centore R., Peluso A., Nejrotti S., Barra M. (2024). Asymmetrical Diketopyrrolopyrrole Derivatives with Improved Solubility and Balanced Charge Transport Properties. Molecules.

[48] Frisch M.J., Trucks G.W. (2016). Gaussian 16, Revision, A.03.

[49] Miertuš S., Scrocco E., Tomasi J. (1981). Electrostatic interaction of a solute with a continuum. A direct utilizaion of AB initio molecular potentials for the prevision of solvent effects. Chem. Phys..

[50] Paternò G.M., Barbero N., Galliano S., Barolo C., Lanzani G., Scotognella F., Borrelli R. (2018). Excited state photophysics of squaraine dyes for photovoltaic applications: An alternative deactivation scenario. J. Mater. Chem. C.

[51] Padula D., Landi A., Prampolini G. (2023). Assessing alkyl side chain effects on electron transport properties of Y6-derived non-fullerene acceptors. Energy Adv..

[52] Chiarella F., Toccoli T., Barra M., Aversa L., Ciccullo F., Tatti R., Verucchi R., Iannotta S., Cassinese A. (2014). High mobility *n*-type organic thin-film transistors deposited at room temperature by supersonic molecular beam deposition. Appl. Phys. Lett..

