# Supplementary material for: Novel Isoindigo-Based Organic Semiconductors End Capped with 1,1-Dicyanomethylene-3-Indanone: Effect of the Bromination and Position of Bromine Substituents on the Chemical–Physical and Electrical Properties
Source: Molecules. 2025 Sep 9;30(18):3672. doi: 10.3390/molecules30183672 (PMC12472438; doi:10.3390/molecules30183672)
Supplement: Supplementary file 1 [file molecules-30-03672-s001.zip › molecules-3809873-supplementary.pdf]

Supplementary materials

Title: "Novel isoindigo based organic semiconductors end capped with 1,1-Dicyanomethylene-3-Indanone: effect of the bromination and of the position of bromine substituents on the chemical physical and electrical properties

Authors: Fabio Mocerino, [a] Mario Barra, [b] Fabio Borbone, [a] Antonio Carella, [a*] Roberto Centore, [a] Fabio Chiarella, [b] Alessandro Landi, [c] Andrea Peluso. [c]

a) Dipartimento di Scienze Chimiche, Università degli Studi di Napoli Federico II, Complesso Universitario di Monte Sant'Angelo, via Cintia 21, 80126, Napoli (Italy); b) CNR-Institute for Superconductors, Innovative Materials and Devices (SPIN, Piazzale Tecchio 80, 80125, Napoli (Italy); c) Dipartimento di Chimica e Biologia, Università di Salerno, Via Giovanni Paolo, 84084, Fisciano (Italy)

Corresponding author: antonio.carella@unina.it

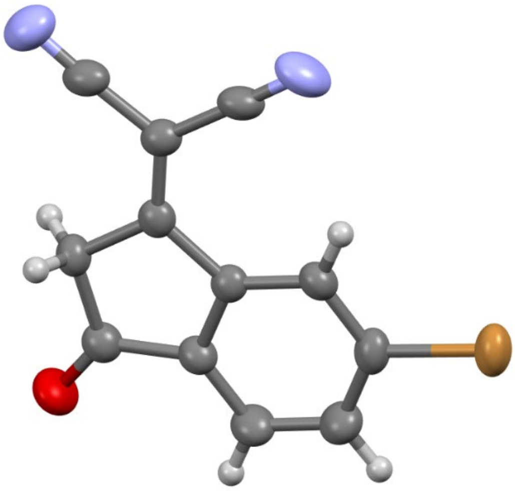

Figure S1. ORTEP diagram of **IDM-6** at 293 K (the ellipsoids are drawn at the 50% probability level)

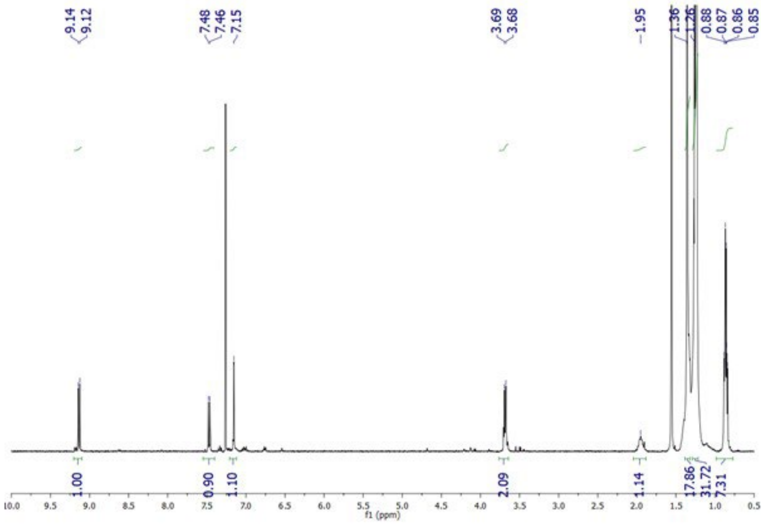

Figure S2. 1H NMR of **II-B** in $CDCl_3$. Signals relative to solvents are starred.

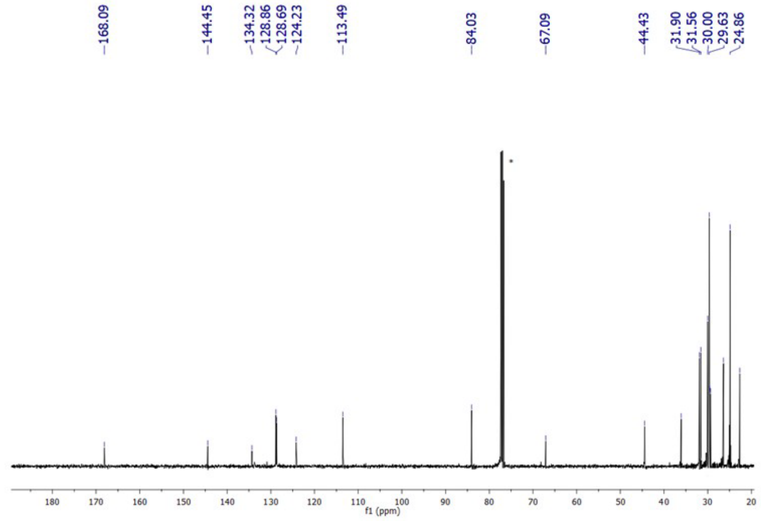

Figure S3. 13C NMR of **II-B** in $CDCl_3$. Signals relative to solvents are starred

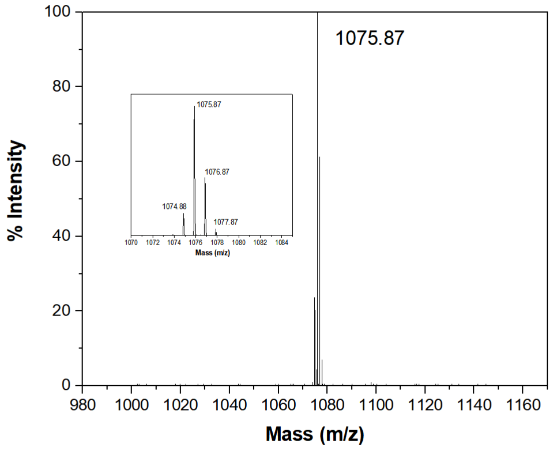

Figure S4. MALDI-TOF mass spectrum of **II-B** (main peak corresponding to $[M+H]^+$).

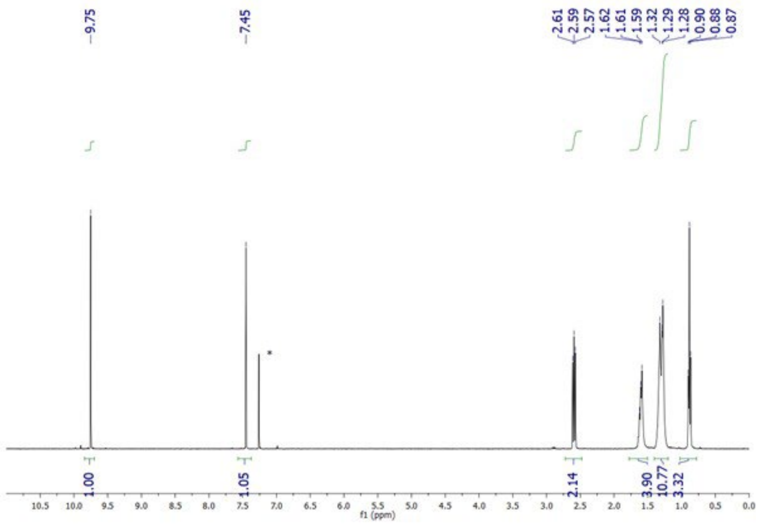

Figure S5. 1H NMR of **T8-CHO**. Signals relative to solvents are starred.

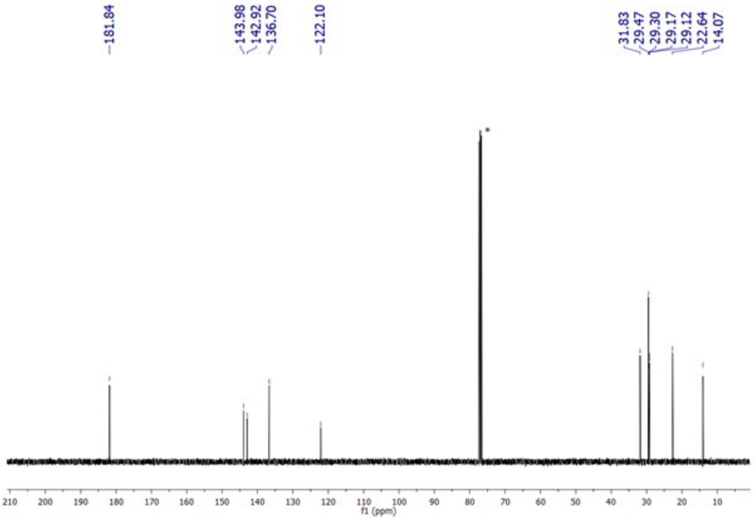

Figure S6. 13C NMR of **T8-CHO** in $CDCl_3$. Signals relative to solvents are starred.

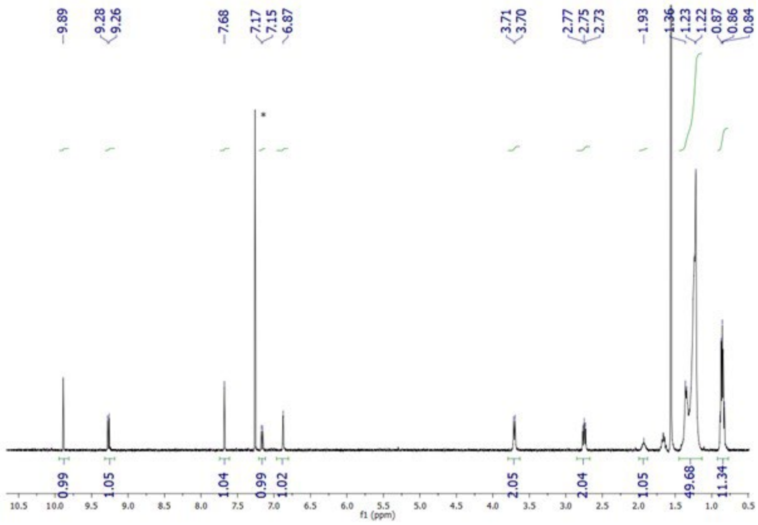

Figure S7. 1H NMR of **II-T8-CHO** in $CDCl_3$. Signals relative to solvents are starred.

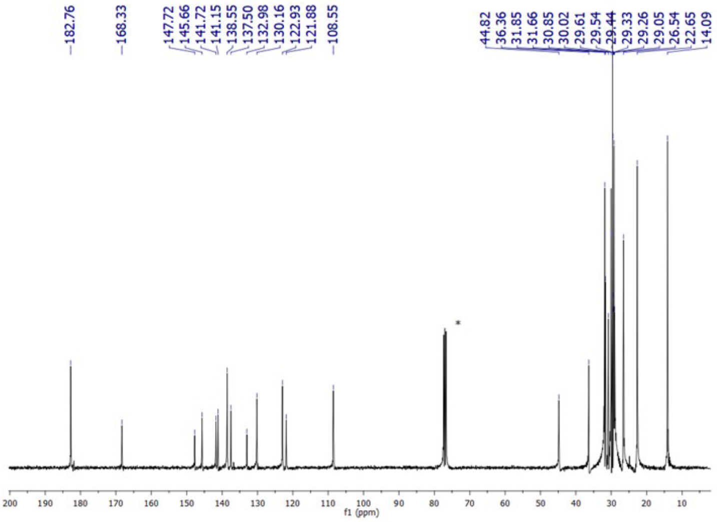

Figure S8. 13C NMR of **II-T8-CHO** in $CDCl_3$. Signals relative to solvents are starred

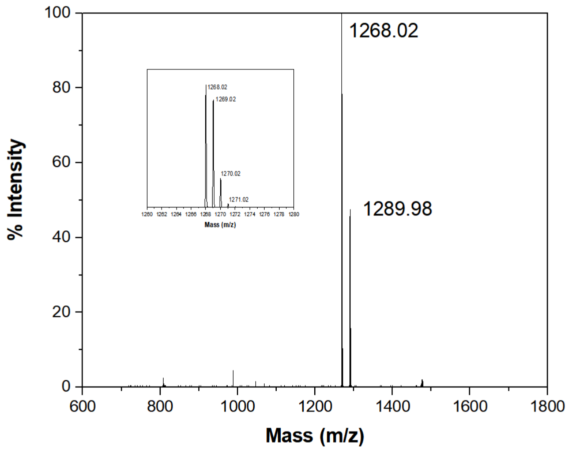

Figure S9. MALDI-TOF mass spectrum **II-T8-CHO** (main peak corresponding to $[M+H]^+$).

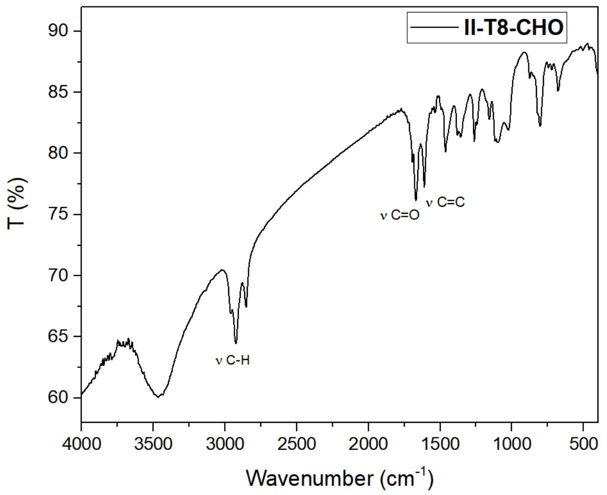

Figure S10. FTIR spectrum for the compound **II-T8-CHO** dispersed in KBr pellet.

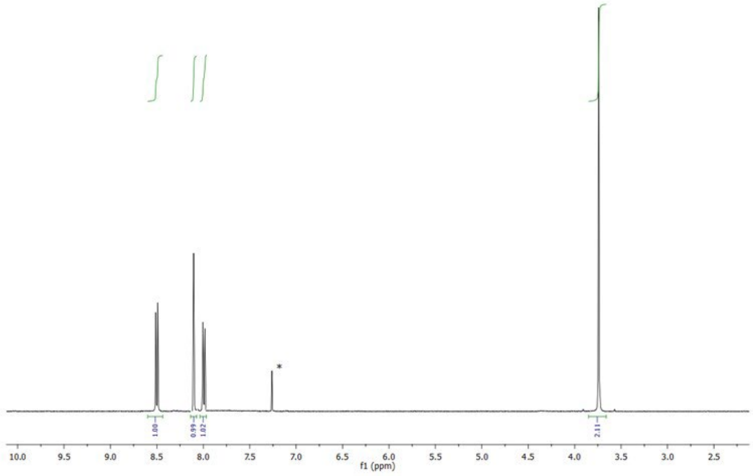

Figure S11. 1H NMR of **IDM-5** in $CDCl_3$. Signals relative to solvents are starred.

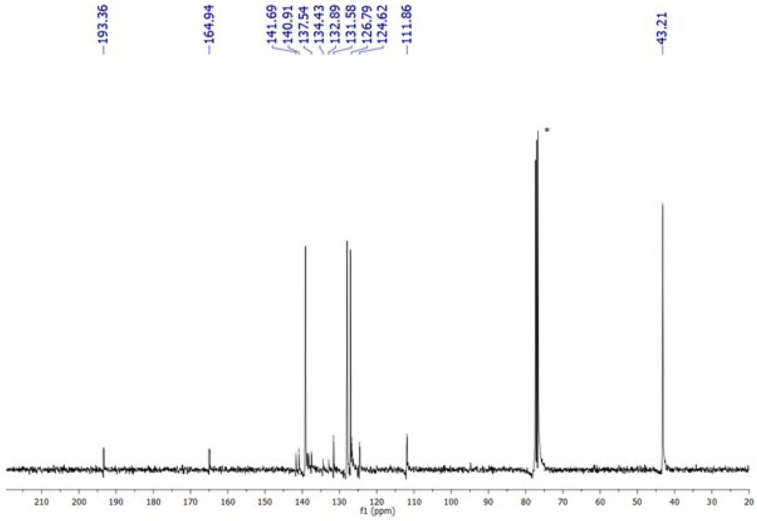

Figure S12. 13C NMR of **IDM-5** in $CDCl_3$. Signals relative to solvents are starred.

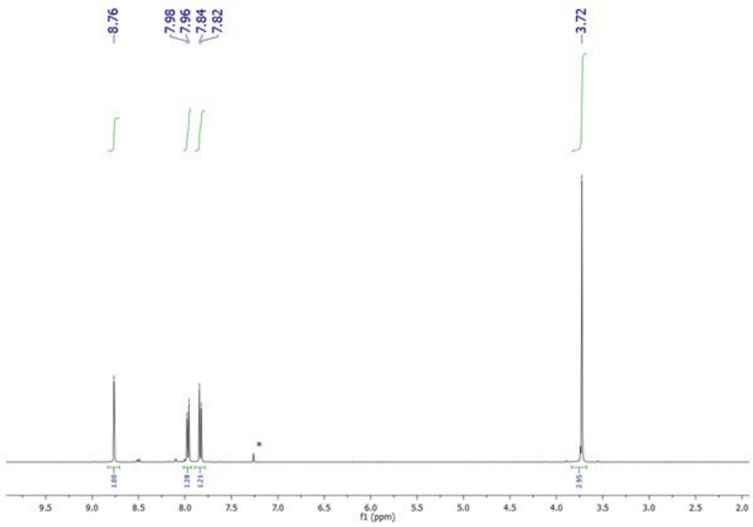

Figure S13. 1H NMR **of IDM-6** in $CDCl_3$. Signals relative to solvents are starred

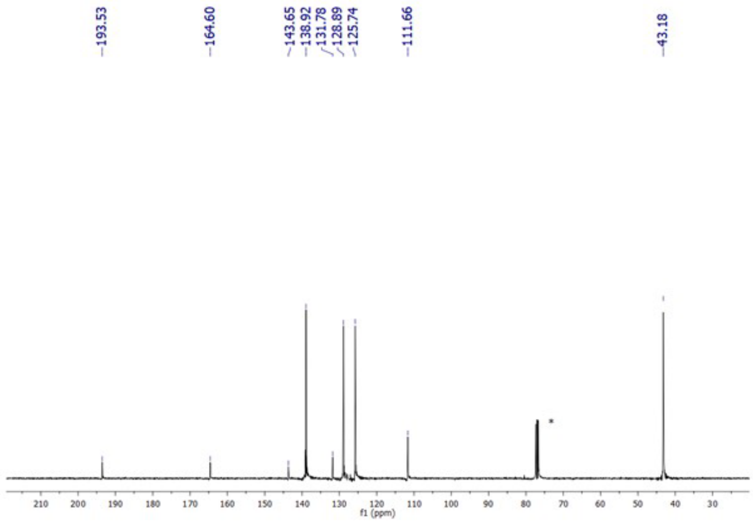

Figure S14. 13C NMR **of IDM-6** in $CDCl_3$. Signals relative to solvents are starred

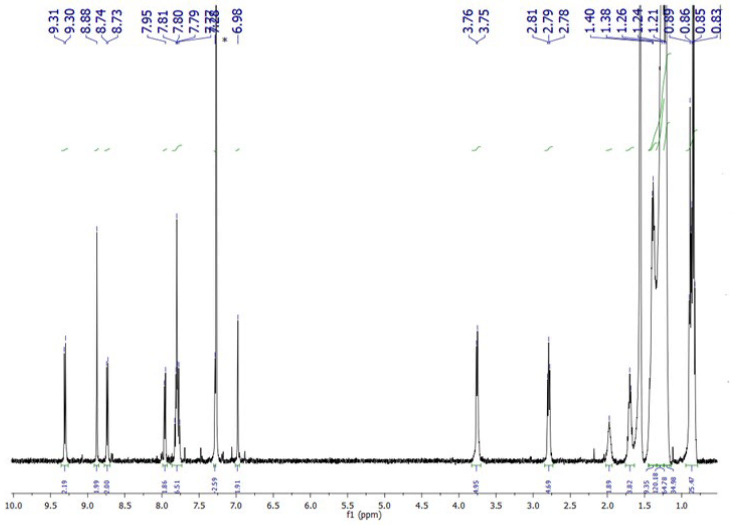

Figure S15. 1H NMR **II-T8-IDM** in $CDCl_3$. Signals relative to solvents are starred.

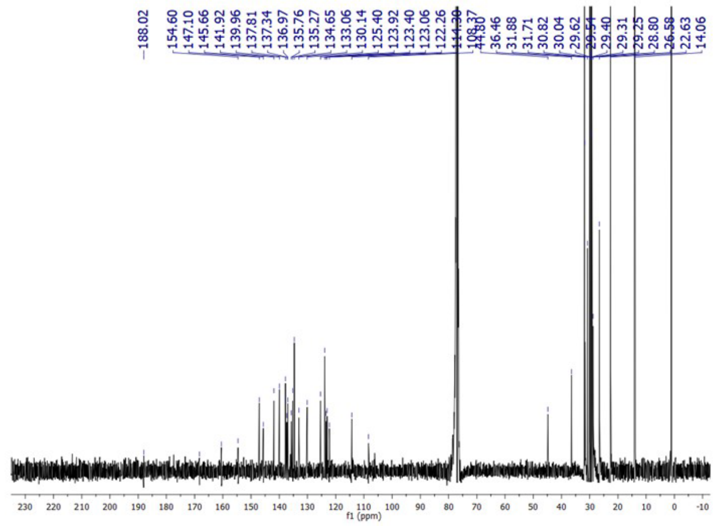

Figure S16. 13C NMR **II-T8-IDM** in $CDCl_3$. Signals relative to solvents are starred

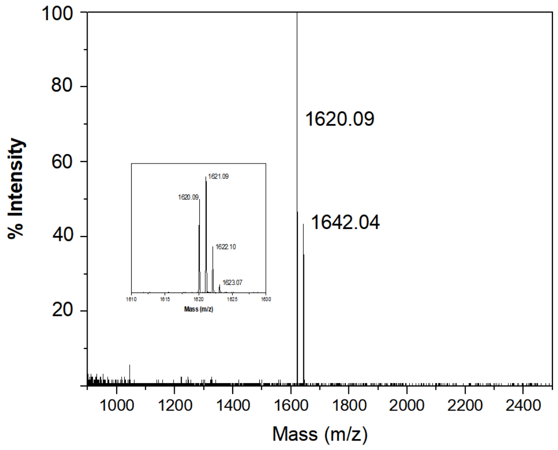

Figure S17. MALDI-TOF mass spectrum of **II-T8-IDM**(main peak corresponding to $[M+H]^+$; a second peak corresponding to $[M+Na]^+$ is observed as well).

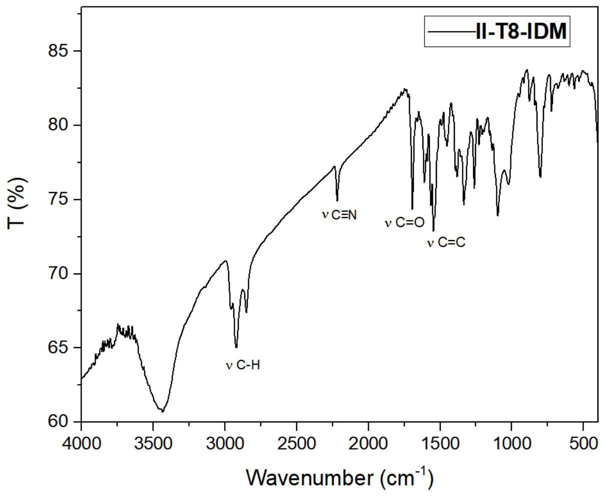

Figure S18. FTIR spectrum for the compound **II-T8-IDM** dispersed in KBr pellet.

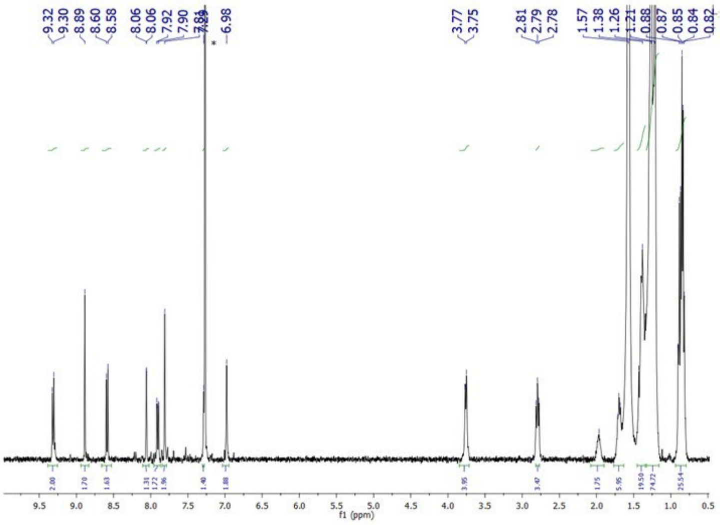

Figure S19. 1H NMR of **II-T8-IDM5** in $CDCl_3$. Signals relative to solvents are starred.

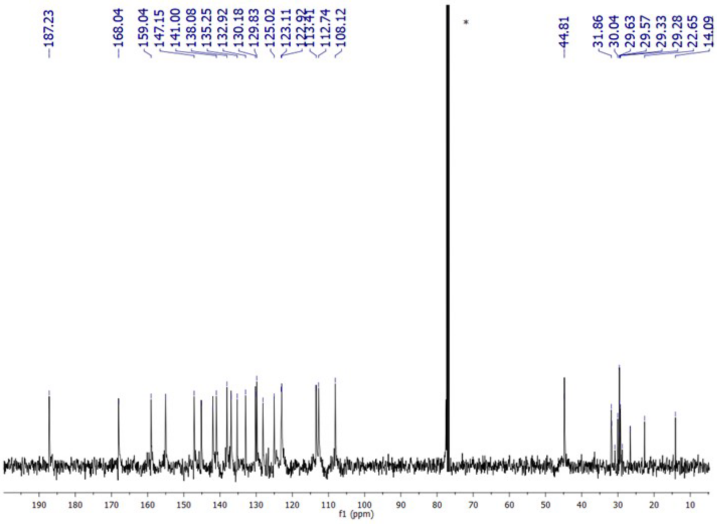

Figure S20. 13C NMR of **II-T8-IDM5** in $CDCl_3$. Signals relative to solvents are starred.

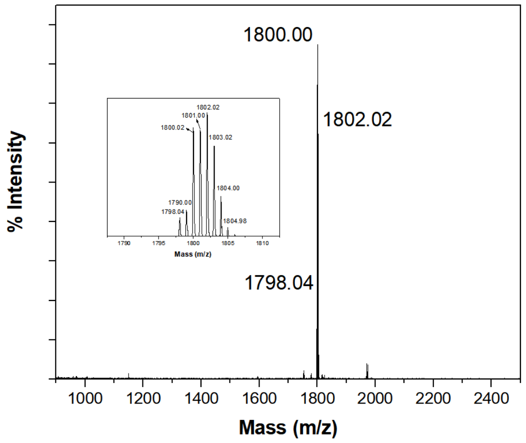

Figure S21. MALDI-TOF mass spectrum of **II-T8-IDM5** (main peak corresponding to $[M+Na]^+$).

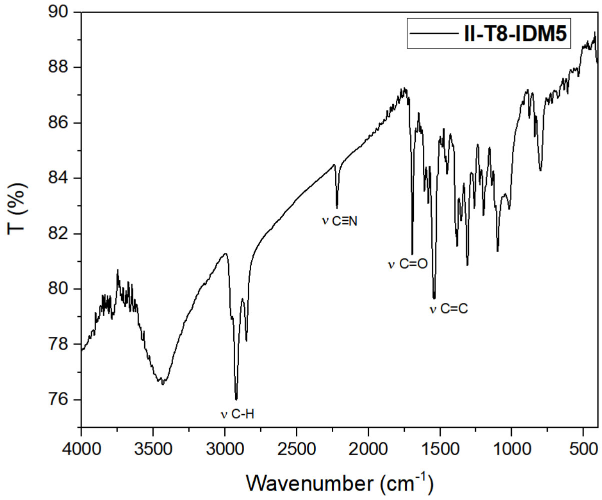

Figure S22. FTIR spectrum for the compound **II-T8-IDM5** dispersed in KBr pellet.

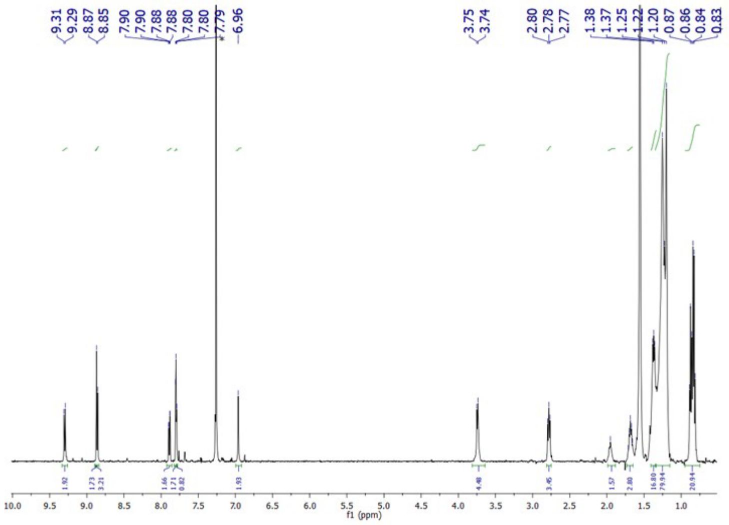

Figure S23. 1H NMR of **II-T8-IDM6** in $CDCl_3$. Signals relative to solvents are starred.

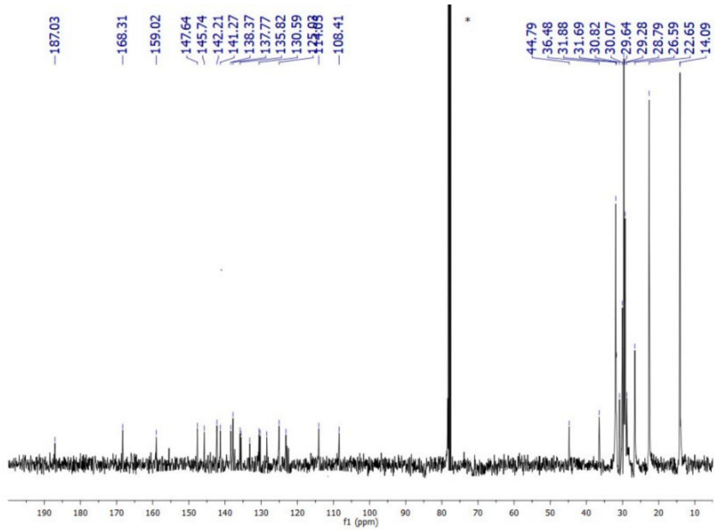

Figure S24. 13C NMR of **II-T8-IDM6** in $CDCl_3$. Signals relative to solvents are starred

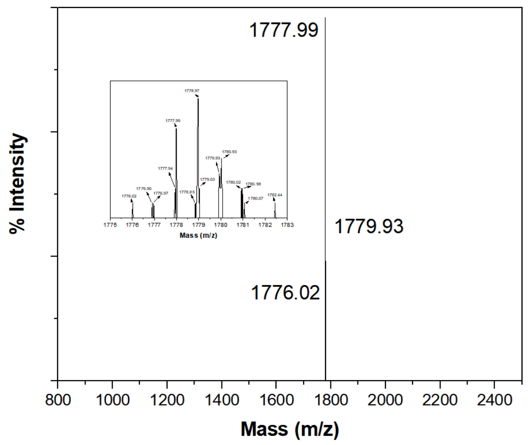

Figure S25. MALDI-TOF mass spectrum of **II-T8-IDM6** (main peak corresponding to $[M+H]^+$).

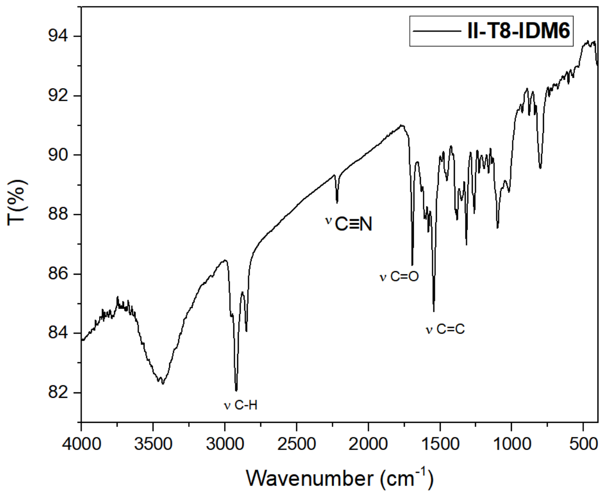

Figure S26. FTIR spectrum for the compound **II-T8-IDM6** dispersed in KBr pellet.

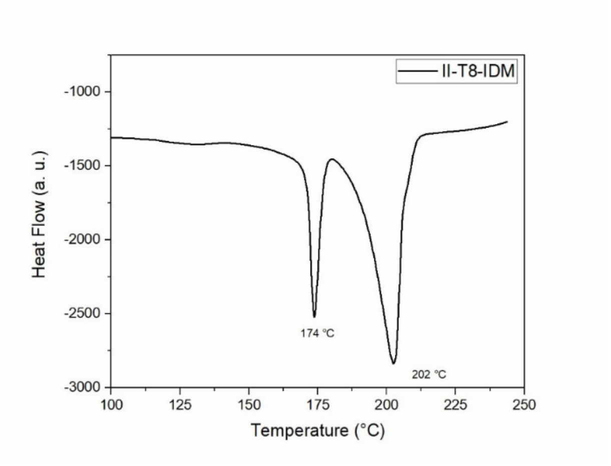

Figure S27. DSC graph of the compound **II-T8-IDM** (run performed in N_2 atmosphere at 10°/min)

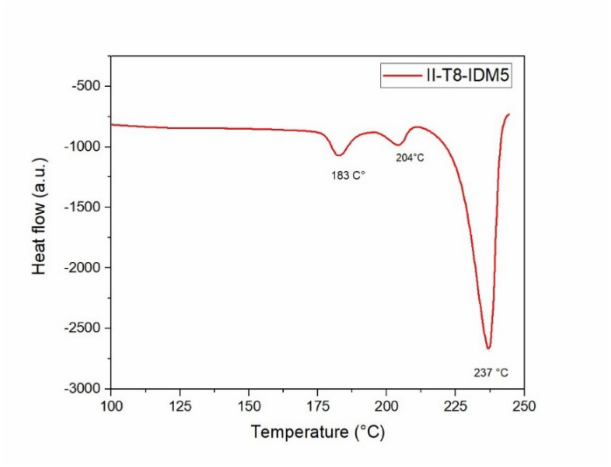

Figure S28. DSC graph of the compound **II-T8-IDM5** (run performed in N_2 atmosphere at 10 °C/min)

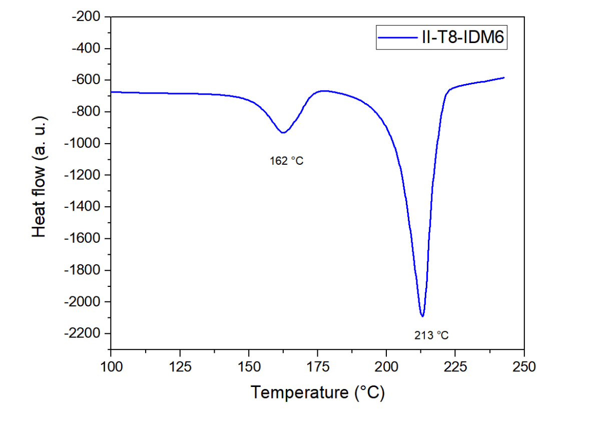

Figure S29. DSC graph of the compound **II-T8-IDM6** (run performed in N_2 atmosphere at 10 °C/min)

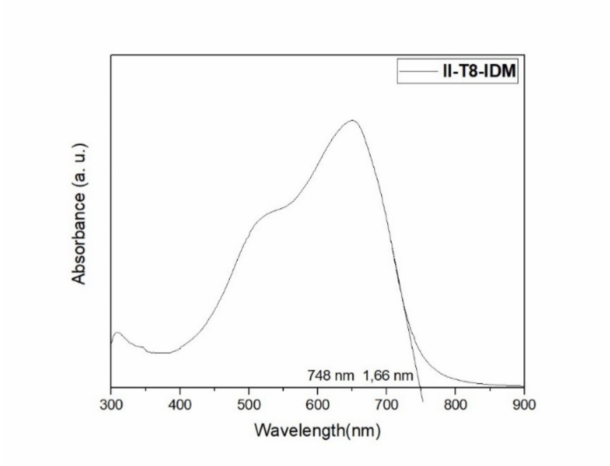

Figure S30. Optical bandgap of **II-T8-IDM** graphically determined from UV-Vis absorption spectrum of a thin film.

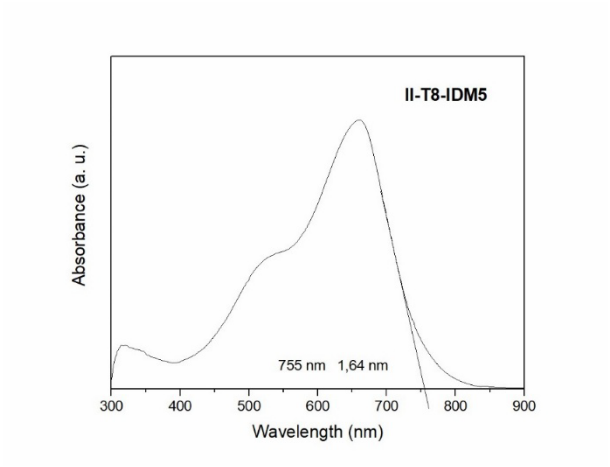

Figure S31. Optical bandgap of **II-T8-IDM5** graphically determined from UV-Vis absorption spectrum of a thin film.

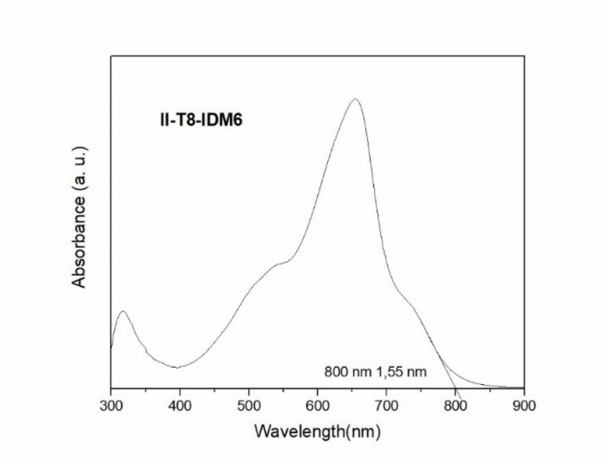

Figure S32. Optical bandgap of **II-T8-IDM6** graphically determined from UV-Vis absorption spectrum of a thin film.

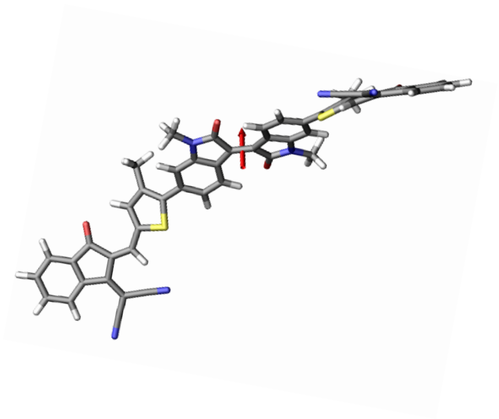

Figure S33. Optimized structure (ground state) for **II-T8-IDM**; the red arrow represents the orientation of the electric dipole moment. Total energy (ZPE corrected): -3497.629315 hartree

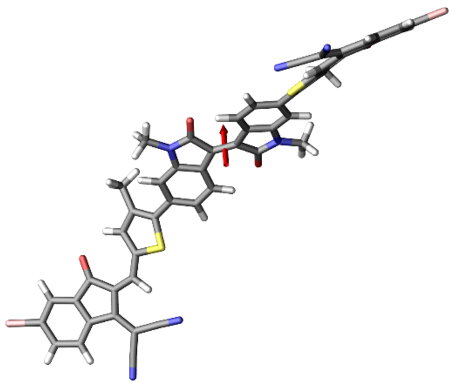

Figure S34. Optimized structure (ground state) for **II-T8-IDM5**; the red arrow represents the orientation of the electric dipole moment. Total energy (ZPE corrected): -8639.257458 hartree

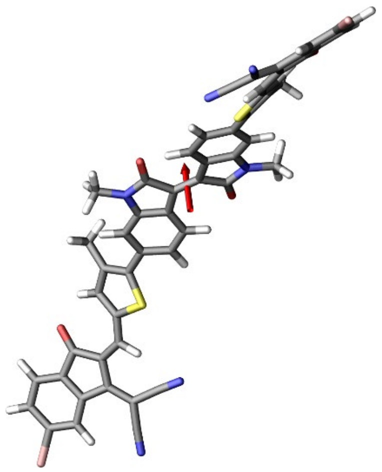

Figure S36. Optimized structure (ground state) for **II-T8-IDM6**; the red arrow represents the orientation of the electric dipole moment. Total energy (ZPE corrected): -8639.256776 hartree

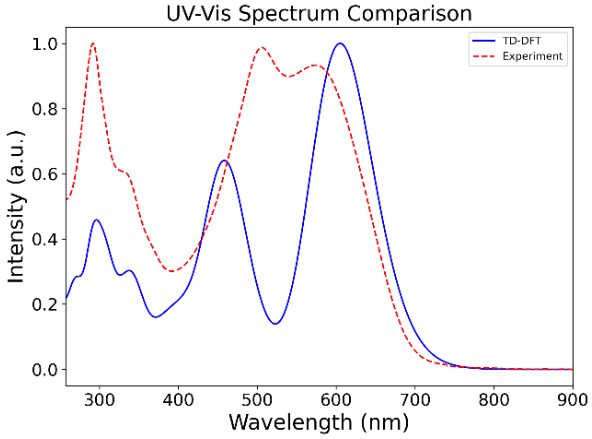

Figure S36. Comparison between the computed (blue full line) and experimental (red dashed line) UV spectra for **II-T8-IDM**.

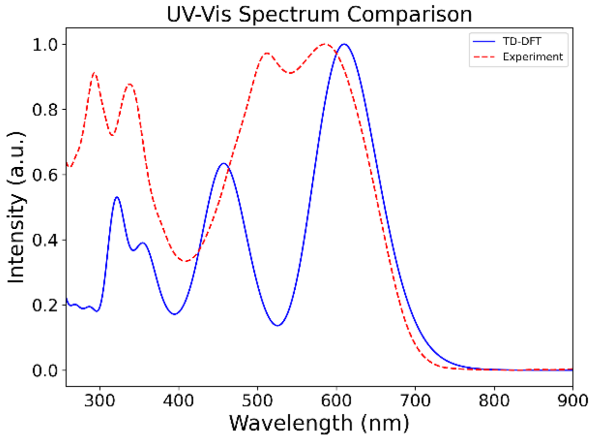

Figure S37. Comparison between the computed (blue full line) and experimental (red dashed line) UV spectra for **II-T8-IDM5**.

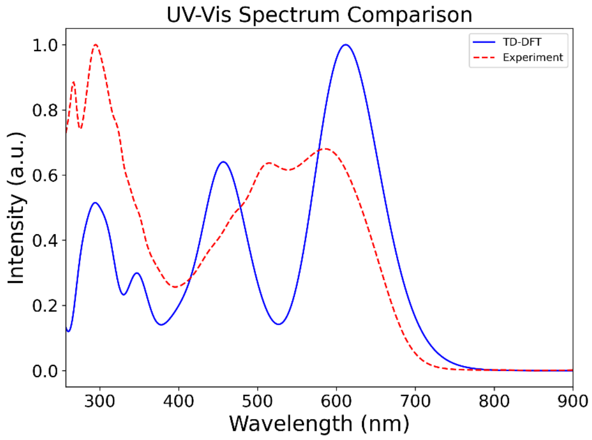

Figure S38. Comparison between the computed (blue full line) and experimental (red dashed line) UV spectra for **II-T8-IDM6**.

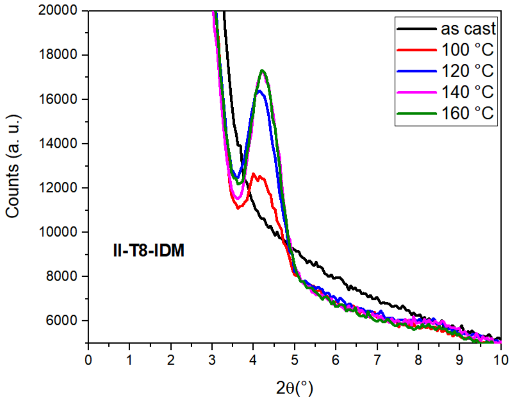

Figure S39. XRD diffraction spectra of thin films of the compound **II-T8-IDM** annealed for 1 h at different temperatures.

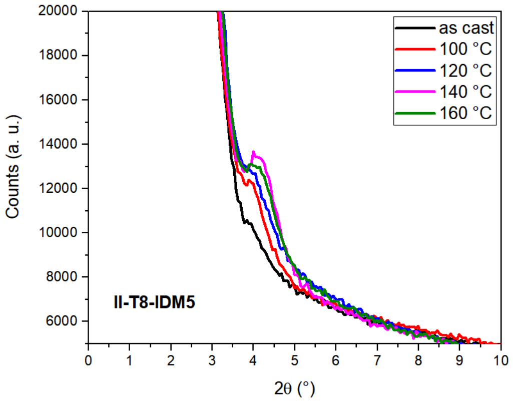

Figure S40. XRD diffraction spectra of thin films of the compound **II-T8-IDM5** annealed for 1 h at different temperatures.

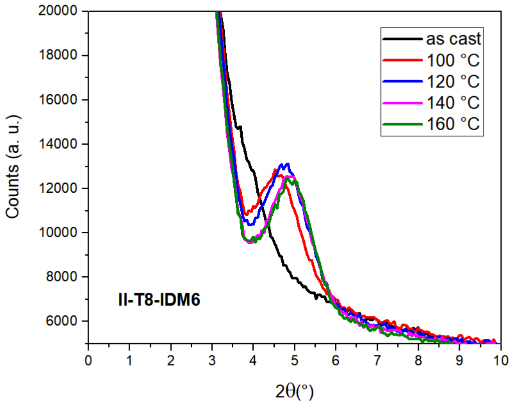

Figure S41. XRD diffraction spectra of thin films of the compound **II-T8-IDM6** annealed for 1 h at different temperatures.

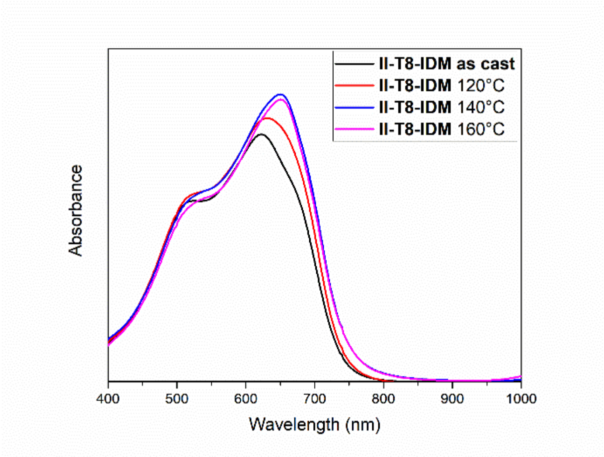

Figure S42. Optical spectra of thin films of the compound **II-T8-IDM** annealed for 1 h at different temperatures.

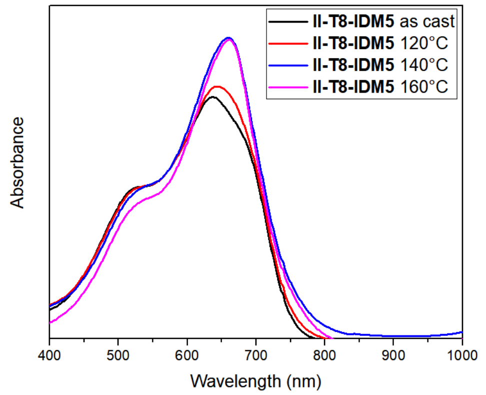

Figure S43. Optical spectra of thin films of the compound **II-T8-IDM5** annealed for 1 h at different temperatures.

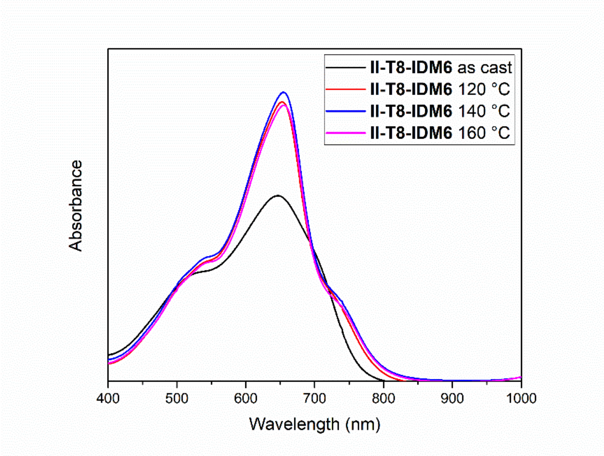

Figure S44. Optical spectra of thin films of the compound **II-T8-IDM6** annealed for 1 h at different temperatures.

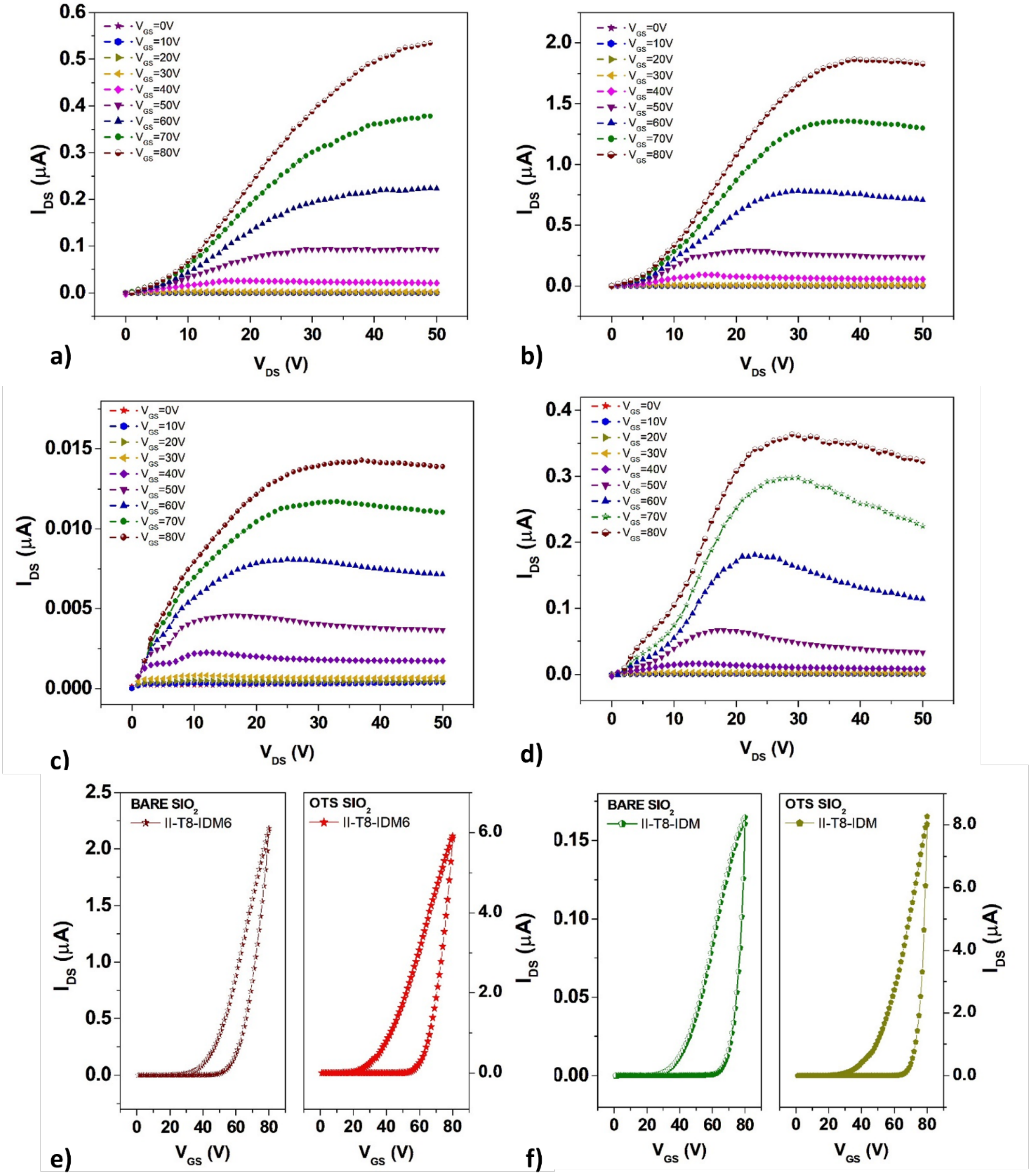

Figure S45. Output curves measured for (a,b)**II-T8-IDM6** and (c,d) **II-T8-IDM** transistors fabricated, respectively, on bare (a, c) and OTS-treated (b, d) SiO_2 substrates. (e,f) Set of transfer curves acquires for (e) **II-T8-IDM6** and (f) **II-T8-IDM** transistors both on bare and OTS-treated SiO_2 substrates

Table S1 Crystal data and structure refinement details for **IDM-6**.

	**IDM-6**
CCDC number	2470625
Empirical formula	$C_{12}H_5BrN_2O$
Formula weight	273.09
T (K)	293(2)
λ (Å)	0.71073
Crystal system	Monoclinic
Space group	*C2/c*
a (Å)	23.400 (1)
b (Å)	5.446 (1)
c (Å)	16.849 (1)
α (°)	90
β (°)	90.19 (4)
γ (°)	90
Volume (Å^3)	2147.2 (4)
Z	8
ρ_{calc} (g/cm^3)	1.690
μ (mm^{-1})	3.805
F(000)	1072.0
2θ range (°)	5.95 – 55
Reflections collected / unique [R(int)]	5226/2310 [0.0390]
Data/restraints/parameters	2310/0/153
Goodness-of-fit on F^2	1.117
Final*R1*, *wR2* indices [I>2σ(I)]	0.0500, 0.1087
Final *R1*, *wR2* indices (all data)	0.0731, 0.1179
Largest diff. peak / hole (eA^{-3})	0.82/-0.61

Table S2. Melting point and solid-solid transition temperatures for the reported **II** based OSCs. The DSC run was conducted in nitrogen atmosphere at scan rate of 10 °C/min

Dye	Melting point (°C)	$T_{k\text{-}k}^{1}$ (°C)	$T_{k\text{-}k}^{2}$ (°C)
**II-T8-IDM**	202	174	
**II-T8-IDM5**	237	183	204
**II-T8-IDM6**	213	162	

Table S3. Computed main optical transitions in the synthesized dyes. Only transitions with Oscillator strength > 0.15 have been reported

Dye	λ_{abs} (nm)	Osc.strength
**II-T8-IDM**	637	1.97
	490	0.73
	466	0.35
	423	0.16
	326	0.25
	320	0.15
	306	0.31
	285	0.20
**II-T8-IDM5**	641	2.06
	494	0.62
	470	0.52
	375	0.22
	340	0.52
	331	0.21
	281	0.15
	264	0.25
**II-T8-IDM6**	644	2.06
	496	0.55
	471	0.58
	432	0.20
	367	0.21
	331	0.19
	328	0.18
	311	0.29
	305	0.21
	290	0.37
	264	0.17
